# Exogenous application of α-Ionone and Methyl jasmonate induces metabolite-based response in *Alstroemeria* and reduces feeding damage by Western Flower Thrips

**DOI:** 10.1371/journal.pone.0355314

**Published:** 2026-08-11

**Authors:** Andrés F. Velasco-Cárdenas, Daniel Rodríguez, Ericsson Coy-Barrera

**Affiliations:** 1 Biological Control Laboratory, Facultad de Ciencias Básicas y Aplicadas, Universidad Militar Nueva Granada, Cajicá, Colombia; 2 Bioorganic Chemistry Laboratory, Facultad de Ciencias Básicas y Aplicadas, Universidad Militar Nueva Granada, Cajicá, Colombia; University of Florida Tropical Research and Education Center, UNITED STATES OF AMERICA

## Abstract

Western Flower Thrips (WFT) (*Frankliniella occidentalis*) is a major pest threatening ornamental floriculture, including *Alstroemeria* production. Conventional insecticide-based management is increasingly limited due to the development of resistance and concerns over non-target impacts. Plant defense elicitors such as methyl jasmonate (MeJA) and α-ionone (α-I) represent promising alternatives for priming endogenous metabolic pathways that deter herbivory. However, their roles in metabolite-related *Alstroemeria* defense activation and associated effects on detoxification-related enzyme activity in thrips remain insufficiently characterized. Thus, exogenous application of defense elicitors in *Alstroemeria* resulted in consistent reductions in thrips florivory across concentrations and time points, with MeJA at 300 ppm and α-I at 150 ppm showing the strongest and most reproducible antifeedant effects. Thrips feeding on elicitor‑treated tissues exhibited clear, treatment‑dependent variation of detoxification enzyme activity, including glutathione *S*‑transferases, esterases, and cytochrome P450 monooxygenases. In parallel, untargeted metabolomic analyses clearly distinguished elicitor‑treated plants from controls and revealed pronounced time‑ and tissue‑specific metabolic reprogramming, particularly reflected in the accumulation of phenylpropanoid‑ and flavonoid‑related features. Collectively, these results demonstrate that MeJA and α-I effectively enhance defense responses in *Alstroemeria*, plausibly constraining WFT feeding. The concordant patterns observed in florivory, insect enzyme activity, and plant metabolite-based responses support the view that elicitor‑driven induction of plant‑derived metabolites contributes to antifeedant effects while simultaneously reshaping dynamics of WFT detoxification enzyme activity. These findings highlight MeJA and α-I as viable elicitors that merit further investigation as components of sustainable thrips management.

## Introduction

The floriculture industry plays a central role in Colombia’s agricultural economy, and *Alstroemeria* is among its most commercially valuable ornamental crops [1,2]. However, *Alstroemeria* production is persistently threatened by the Western Flower Thrips (WFT) species complex (*Frankliniella occidentalis*), a polyphagous pest that causes extensive qualitative and quantitative damage to floral tissues [3,4]. Thrips feeding punctures disrupt pigmentation, reduce aesthetic value, and diminish marketability, while their rapid reproductive cycles and high dispersal ability facilitate establishment in greenhouse environments [4]. Conventional management relies heavily on synthetic insecticides, yet the recurrent emergence of insecticide resistance [5–7] and the documented non‑target impacts on beneficial arthropods [8–11] challenge the long-term effectiveness and ecological sustainability of these approaches. These limitations highlight an urgent need for alternative or complementary strategies that enhance plant-mediated resilience against thrips.

Plants possess sophisticated defense systems composed of constitutive and inducible chemical mechanisms that deter herbivory or interfere with herbivore physiology [12]. Inducible defenses, in particular, are regulated by signaling pathways mediated by jasmonates and apocarotenoid-derived compounds, which can activate downstream metabolic responses [13,14]. These pathways promote the accumulation of specialized metabolites, including phenylpropanoids, flavonoids, and related compound classes, that can reduce herbivore performance, influence feeding behavior, or disrupt detoxification processes [13,15]. Exogenous application of defense elicitors has therefore emerged as a promising strategy to activate inducible defense pathways without genetic modification, thereby enabling targeted priming of plant tissues to anticipate herbivory [13,16]. Among known elicitors, methyl jasmonate (MeJA) is well established as a regulator of herbivore-responsive secondary metabolism, leading to the induction of phenolics, volatile organic compounds, and defense-related metabolites [14,17]. α-Ionone (α-I), a volatile apocarotenoid, has also been shown to influence plant–insect interactions, including the induction of resistance to WFT, although its role in ornamental crops and the underlying mechanisms remain comparatively less explored [18].

Plant defense priming via elicitor application is a promising, eco-friendly strategy for activating inducible defense pathways and enhancing resistance [13,16,19]. The elicited accumulation of specialized metabolites with lethal and sublethal properties can, in some cases, impair insect detoxification enzymes, e.g., by reducing glutathione *S*-transferase (GST) and cytochrome P450 activities, thereby compromising herbivore tolerance [15]. Sustained feeding under impaired detoxification capacity may result in intoxication and reduced herbivore performance [15,20]. Compared to broad-spectrum insecticides, defense elicitation generally poses lower risks to beneficial arthropods, including pollinators and predators [9,21]. Despite growing interest in elicitor-based pest management, the activation of induced chemical defenses in *Alstroemeria* and their consequences for thrips physiology have received limited empirical attention. In particular, it remains unclear whether elicitor-mediated metabolic reprogramming in this ornamental species translates into biologically meaningful reductions in thrips feeding and whether such changes are associated with alterations in insect detoxification enzyme activity [16]. Addressing these knowledge gaps is critical for evaluating the relevance of elicitor-based strategies in floriculture systems.

In this context, the present study aimed to investigate the influence of exogenous MeJA and α-I on defense activation in *Alstroemeria* and the effects of this inducible plant-derived response on WFT feeding behavior and detoxification biochemistry. Specifically, we assessed whether: (i) exogenous elicitor treatments reduce florivory; (ii) thrips feeding on elicitor-treated tissues exhibit changes in GST, esterase, and cytochrome P450 activities; and (iii) elicitor application induces measurable shifts in the metabolite profiles of leaf and tepal tissues, as characterized through untargeted LC–MS metabolomics. By integrating phenotypic, biochemical, and metabolomic approaches, we explored the potential of plant defense elicitors as tools for sustainable thrips management in ornamental production systems.

## Materials and methods

### Experimental overview

This study evaluated whether exogenous application of two plant defense elicitors (i.e., methyl jasmonate (MeJA) and α‑ionone (α-I)) enhanced chemical defenses in *Alstroemeria* and reduced feeding damage caused by Western Flower Thrips (WFT). The experimental approach integrated three components: (i) elicitor treatments on greenhouse-grown plants; (ii) assessments of thrips florivory and detoxification enzyme activity; and (iii) untargeted metabolomics-based variation of leaf and tepal tissues at multiple time points following elicitation. Elicitor treatments were applied at two concentrations (150 and 300 ppm), and the responses were evaluated at 48, 96, and 144 h post-application. Each treatment–timepoint combination included six independent biological replicates. Experimental steps were conducted in a randomized arrangement to minimize positional or temporal bias. Considering the biological material was obtained from private, managed agricultural facilities and does not involve native species or protected sampling areas, a formal collection or research permit from environmental authorities is not applicable.

### Plant material and growth conditions

*Alstroemeria* spp. var. ‘Doris’ plants were cultivated under greenhouse conditions in Cajicá, Colombia (2,558 masl; coordinates: 4°56’33’‘N 74°00’46’‘W). The location is classified as having a subtropical highland climate (Cfb) under the Köpen-Geiger climate classification system. To avoid transplantation stress, plants completed their entire developmental cycle in 15-L black polyethylene grow bags filled with silt loam soil (pH 6.4; organic matter 5.2%) and fertilized every 3 weeks with Agrimins® and NPK 15-15-15. Irrigation (2 L/day) was automated. Environmental greenhouse conditions during growth averaged 26 ± 8 °C, 65 ± 20% relative humidity (RH), and photosynthetically active radiation (PAR) 420–780 µmol m^–2^ s^–1^ at canopy height under a 12:12-h photoperiod (light:dark), mimicking equatorial production zones (e.g., Colombia). Plants were maintained insect-free with protective netting until they reached uniform anthesis and flowering. Only plants exhibiting comparable growth and floral development were used in trials to reduce phenotypic variability.

### Insect rearing and maintenance

A colony of *F. occidentalis* was maintained for more than 10 generations under controlled environmental conditions (20 ± 5 °C, 70 ± 10% RH, 12L:12D). Thrips were reared on red clover flowers (*Trifolium pratense*). Adult thrips used in experiments were collected from the colony and maintained without exposure to insecticides. Prior to bioassays, adult thrips were sieved to obtain cohorts of 24–48 h adults. Preliminary screening confirmed sex ratios of approximately 65:35 (female:male). Species identification followed established morphological criteria [22–24].

### Elicitor treatments

Solutions of MeJA and α-I (≥95% purity; Sigma-Aldrich) were separately prepared at 150 and 300 ppm in sterile distilled water (SDW) containing 0.1% Tween-20 (T20) to ensure droplet adhesion. This solvent mixture (i.e., SDW with 0.1% T20) served as the untreated control (CW). The concentrations evaluated in this study (150 and 300 ppm) were selected based on established thresholds reported in the literature. For MeJA, the maximum dose of 300 ppm (~1.34 mM) aligns with effective anti-herbivore defenses documented at 1–3.55 mM in crops like strawberry [25] and cotton [26]. For α-I, while prior studies reported mild anti-thrips effects at lower doses of 150–300 µM [18], we employed higher concentrations of 150 and 300 ppm (780 and 1560 µM) to guarantee a robust, active physiological response to both elicitors. Treatments were applied to aerial tissues using a handheld atomizer, ensuring full coverage of leaves and inflorescences. Plants (n = 72) were arranged in a factorial design composed of treatment × time, with six biological replicates per treatment. Evaluations were performed at 48, 96, and 144 h post-elicitor application (p-ea).

### Florivory bioassay

After completing the host-plant elicitation phases at 48, 96, and 144 h, the subsequent herbivory trials were conducted using adult thrips of uniform age. Florivory was quantified using excised tepals. Twenty adult thrips were placed in 15‑cm Petri dishes and allowed to feed on freshly collected tepals for 24 h. Six biological replicates per treatment were analyzed at each time point (i.e., 48, 96, and 144 h p-ea). To address the potential impact of excision on food quality, preliminary laboratory trials were conducted to assess tissue stability under the experimental conditions. These validations confirmed that the detached tepals did not undergo significant deterioration over the 24-h testing period. Furthermore, zero adult thrips mortality was recorded during this 24-h timeframe. Tepals were photographed under standardized illumination and position using a Nikon D3300 digital camera. Digital images were preprocessed in Barvocuc [27] using a standardized protocol to maximize contrast between consumed and intact leaf tissues. The resulting images were then analyzed in ImageJ to quantify the total feeding damage area (cm^2^).

### Enzyme activity assays

To assess the effects of elicitor-induced metabolites on thrips detoxification systems, enzyme activities of GST, esterases (EST), and cytochrome P450 monooxygenases (P450) were measured. Twenty surviving thrips per treatment were pooled and homogenized in 0.1 M sodium phosphate buffer (500 μL, pH 7.2) on ice. Homogenates were centrifuged at 12044 × g for 20 min at 4 °C, and supernatants were used for assays. GST activity was measured using the CDNB substrate at 340 nm, esterase activity using p‑nitrophenyl acetate at 405 nm, and P450 activity using the TMBZ reaction at 630 nm, following the previously reported protocols [28]. Enzymatic assays were performed in 96-well plates, and absorbance was measured using a Varioskan LUX microplate reader. Each enzymatic reaction included blanks without enzyme, a heat-denatured control, and a solvent-only control. Measurements were performed in four technical replicates. Protein concentration was standardized by the Bradford method.

### Metabolite extraction

Leaf and tepal tissues were harvested at 48, 96, and 144 h p-ea, immediately flash-frozen, and stored at –80 °C. Samples were extracted in absolute ethanol (1:1 g/mL). Tissue was bead-disrupted, centrifuged at 8450 × g for 5 min at 4 °C, filtered through 0.22 µm PTFE membranes, and stored at –20 °C until LC–MS analysis.

### LC-MS analysis

Extracts (1 µg/mL; 10 µL injection) were analyzed using a Shimadzu LC-MS2020 system equipped with DAD, ESI, and a quadrupole analyzer. Chromatographic separation was performed on a Kinetex C18 column (4.6 × 150 mm; 2.2 µm) at 0.7 mL/min using 1% formic acid in water and 1% formic acid in acetonitrile as mobile phases A and B, respectively. The gradient elution method involved 0–2 min 5% B, 2–11 min 0 → 50% B, 10–13 min 50% B, 13–17 min 50 → 100% B, 17–19 min 95%, and 19–22 min 95 → 5% B. Detection was simultaneously monitored at 270 nm and under negative-ionization mode. Quality controls included pooled extracts (QCPS) and a multimix reference standard (QCR: quercetin, rutin, gallic acid, olivetol, catechin) to monitor analytical stability. High-resolution MS data were additionally acquired using an Agilent 1260 LC coupled to a Q-ToF system with AJS ESI for feature annotation under consistent chromatographic conditions.

### Raw LC-MS data processing, discriminant analysis, ranking, and feature annotation

Raw LC-MS data were processed in MZmine 3 following standard workflows (feature detection, deconvolution, alignment, normalization) [29]. Features with ≥50% presence across samples and a coefficient of variation <30% in QC samples were retained for analysis. Data matrices were imported into the software SIMCA 14.1 (Umetrics Inc., Umeå, Sweden) for discriminant analysis. Features with variable importance in projection (VIP) > 1 were selected for biological interpretation. Putative metabolite identities were assigned following high‑resolution mass accuracy (≤5 ppm), predicted molecular formulas, MS/MS patterns, retention behavior, adduct patterns, and database matching (e.g., dictionary of natural products, coconut DB, KNApSAcK, PubChem). All metabolite annotations correspond to an HRMS-based confidence level of 3 (tentative candidates that ensure compound class) [30].

### Statistical analysis

Florivory and enzyme activity data were analyzed using ANOVA under a completely randomized design. Normality and homoscedasticity were evaluated using the Shapiro–Wilk and Bartlett tests, respectively. These analyses were performed using the statistical language R version 4.5.2. [31]. On the other hand, metabolite-based differences were explored using orthogonal partial least squares discriminant analysis (OPLS‑DA), with VIP scores (>1) used to identify the top-ranked discriminant features. Data processing included sum normalization and mean centering using SIMCA 14.1 (Umetrics Inc., Umeå, Sweden). The models’ validity was assessed by permutation tests (PT) (200 permutations). OPLS‑DA models were accepted only if Q^2^ > 0.5 and PT showed no overlap between original and permuted distributions.

## Results

### Reduction in thrips florivory after elicitor treatment

Thrips feeding damage on *Alstroemeria* tepals was assessed under controlled laboratory conditions using excised floral tissues after elicitor application. Thrips feeding was evaluated specifically on *Alstroemeria* tepals because this tissue is both a primary target of WFT and a key determinant of commercial quality in ornamental production. In the cultivar ‘Doris’, thrips damage is predominantly observed on floral structures, particularly tepals, where feeding punctures cause visible discoloration, epidermal scarring, and loss of pigmentation. These symptoms directly compromise the aesthetic value of cut flowers and are among the main reasons for market rejection. In addition, thrips feeding responses were assessed using excised tepals to enable precise, standardized, and reproducible quantification of florivory under controlled laboratory conditions. The use of excised floral tissues allows multiple tepals of uniform size, developmental stage, and physiological condition to be exposed simultaneously to thrips feeding, thereby minimizing variability associated with whole‑plant architecture, inflorescence position, or microclimatic heterogeneity.

**Fig 1:**
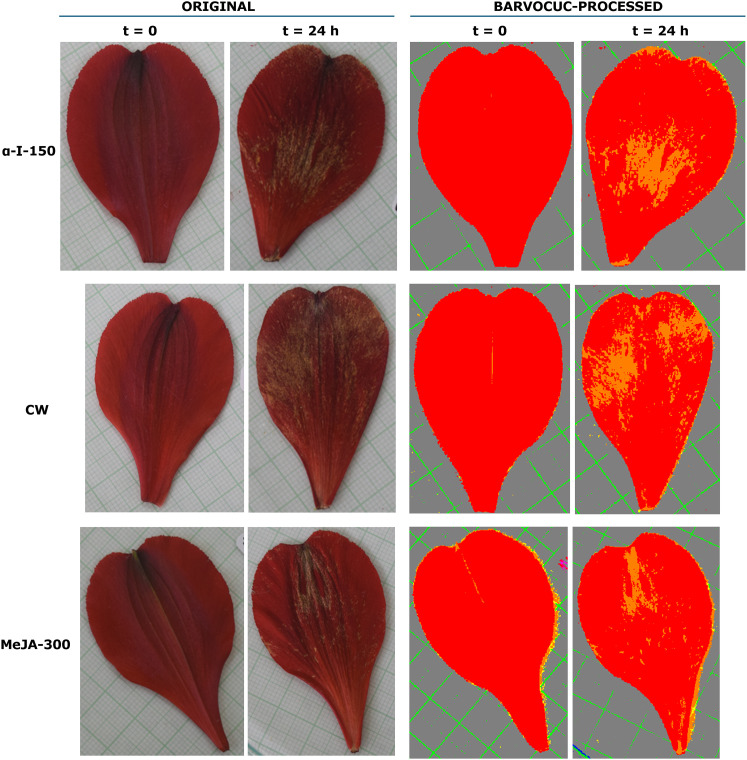
Representative images illustrating thrips florivory on excised *Alstroemeria* tepals for three 48-h post-elicitation treatments: α‑ionone at 150 ppm (α-I‑150; top row), water control (CW; middle row), and methyl jasmonate at 300 ppm (MeJA‑300; bottom row). The assay was initiated 48 h post-elicitation, which corresponds to the baseline (t = 0) of the feeding period. The subsequent time point (t = 24) represents the status after a 24-h period of thrips feeding under laboratory conditions. Original (unprocessed) and Barvocuc‑processed images of *Alstroemeria* tepals are shown. Original (unprocessed) images illustrate the visual appearance of tepals before and after thrips feeding. Barvocuc‑processed images enhance contrast between intact and consumed tissue, with damaged (consumed) areas highlighted in yellow and non‑consumed tissue in red, facilitating subsequent quantification of florivory using ImageJ. Compared with the control, both elicitor treatments reduced thrips feeding after 24 h, with MeJA‑300 showing the most pronounced reduction in the area of consumed tissue. Images are representative of independent biological replicates.

**Fig 2:**
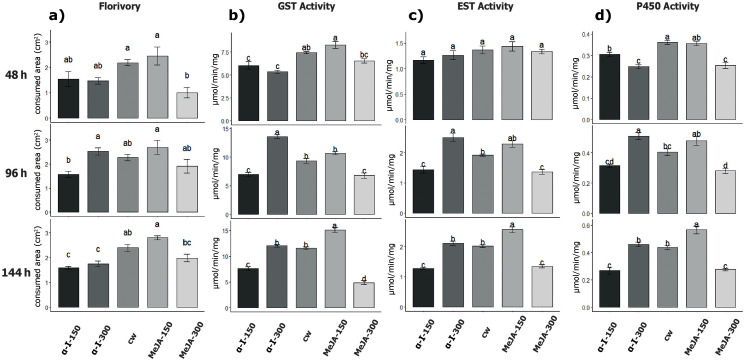
(a) Florivory rate (cm2) and (b, c, f) enzyme activity (µmol/min/mg protein) of (b) GST, (c) esterases, and (d) P450. Each bar represents the mean response for a treatment group: α-ionone (α-I) at 150 ppm (α-I-150), α-I at 300 ppm (α-I-300), control using water (CW), methyl jasmonate (MeJA) at 150 ppm (MeJA-150), and MeJA at 300 ppm (MeJA-300). Different lowercase letters above the bars indicate statistically significant differences among treatment means (n  =  6) within each time point (Tukey’s HSD test, p  <  0.05). The data illustrate both temporal and treatment-dependent variations in florivory and detoxification enzyme activity.

Representative images of tepals collected at 48 h p-ea, recorded at the beginning of the bioassay (t = 0) and after 24 h of exposure to adult thrips, are shown in Fig 1. These bioassays revealed significant variations in florivory intensity among treatments after the feeding period. Specifically, elicitor applications resulted in marked, time-dependent reductions in thrips feeding damage on *Alstroemeria* tissues. Across all evaluations, MeJA at 300 ppm (MeJA‑300) and α‑ionone at 150 ppm (α-I‑150) elicited the strongest antifeedant responses (Fig 1 and Fig 2a).

Qualitatively, in the control treatment (CW) (Fig 1), thrips feeding resulted in extensive tissue damage after 24 h, characterized by irregularly distributed feeding scars and widespread discoloration across the tepal surface. This damage was particularly evident along the central and basal regions of the tepals, consistent with intense florivory typically associated with WFT. In contrast, tepals from plants treated with MeJA‑300 exhibited visibly reduced feeding damage after 24 h, with feeding scars confined to smaller, more localized areas. Similarly, α-I-treated tepals at 150 ppm showed a marked reduction in florivory compared with CW, although scattered feeding patches were still detectable. To facilitate objective quantification of the area of consumed tissue, the original tepal images were preprocessed using Barvocuc software [27] to enhance contrast between damaged and undamaged regions. In the processed images, consumed areas were clearly distinguished from intact tissue, enabling subsequent measurement by digital image analysis. Barvocuc‑processed images (Fig 1) qualitatively confirmed a pronounced reduction in the proportion of consumed area in elicitor‑treated tepals relative to the control. Subsequent quantification in ImageJ confirmed these visual trends by measuring total feeding damage in cm^2^ (Fig 2a).

At 48 h post-elicitor application (p-ea), ANOVA revealed significant differences in consumed areas (cm^2^) among the treatment groups (*p =* 4.5 × 10^–4^). The Tukey HSD post hoc test indicated a significant reduction in florivory rate for the MeJA-300 treatment compared with the control (CW) (*p =* 0.014). At 96 h p-ea, the α-I-150 treatment demonstrated the lowest florivory rate; however, Tukey’s HSD test showed no significant differences between α-I-150 and CW (*p =* 0.16). Notably, α-I-150 differed significantly from both α-I-300 (*p =* 0.027) and MeJA-150 (*p =* 7.0 × 10^–3^). Finally, at 144 h p-ea, significant differences among treatment means were observed (*p =* 4.1 × 10^–7^), with the α-I-150 ppm treatment again showing the lowest florivory rate. Significant differences were found between α-I-150 and α-I-300, and between α-I-150 and CW, using the Tukey HSD test (*p =* 3.2 × 10^–4^ and 4.9 × 10^–3^, respectively). These patterns indicate concentration‑dependent and compound‑specific differences in elicitor performance. MeJA-300 promoted a strong, early-stage suppression of feeding damage at 48 h p-ea, though this protective effect was transient and attenuated over time. Conversely, α-I-elicited resistance followed a delayed trajectory, with its deterrent effect emerging clearly at the 144-h mark.

### Effects of Treatments on Detoxification Enzyme Activity in Thrips

The detoxification enzymes (GST, EST, P450) showed treatment- and time-dependent changes, reflecting WFT’s biochemical response to elicitor-induced chemical defenses in *Alstroemeria* (Fig 2b-d). GST and P450 were the most affected enzymes, exhibiting significant reductions in activity at specific time points.

### Glutathione *S*-transferases (GST)

Significant differences in GST activity were observed across treatments at all post-treatment time points (Fig 2b). At 48 h p-ea, the α-I-300 group exhibited the lowest enzyme activity, which was significantly lower than control (CW) (*p* = 2.5 × 10^–4^; Tukey HSD, *p =* 3.3 × 10^–3^). At 96 h p-ea, significant differences were detected among all treatments (p = 1.5 × 10^–6^), with both α-I-150 and MeJA-300 treatments showing significantly reduced GST activity compared to CW (*p =* 0.012 and 7.6 × 10^–4^, respectively). Interestingly, at this time point, α-I-300 exhibited the highest GST activity, which was significantly higher than that of CW (*p =* 1.7 × 10^–4^). At 144 h p-ea, significant differences were again observed between treatments (*p =* 1.9 × 10^–8^). The MeJA-300 treatment exhibited the lowest GST activity, significantly lower than CW (*p =* 1.0 × 10^–6^), followed by α-I-150 (*p =* 1.2 × 10^–4^). Finally, the MeJA-150 treatment showed the highest activity, significantly exceeding that of CW (*p =* 3.2 × 10^–4^).

### Esterases (EST)

Esterase activity remained unchanged at 48 h p-ea (*p =* 0.19) (Fig 2c). However, by 96 h p-ea, significant differences among all treatments were observed (*p =* 4.1 × 10^–5^). Both α-I-150 and MeJA-300 treatments showed reduced EST activity compared to CW (p = 0.049 and 0.022, respectively). In contrast, the α-I-300-treated group exhibited the highest activity, significantly higher than CW (*p =* 0.016). At 144 h p-ea, significant differences persisted (*p =* 2.3 × 10^–7^), with α-I-150 and MeJA-300-derived groups again presenting the lowest EST activity (*p =* 6.6 × 10^–5^ and 1.6 × 10^–4^, respectively). In contrast, the MeJA-150-treated group exhibited the highest activity, significantly exceeding that of CW (*p =* 9.4 × 10^–4^).

### Cytochrome P450 monooxygenases (P450)

P450 activity was susceptible to treatment across all time points (*p* < 0.05) (Fig 2d). At 48 h p-ea (*p =* 3.8 × 10^–5^), α-I-150, α-I-300, and MeJA-300 treatments reduced P450 activity compared to CW (*p =* 0.033, 1.8 × 10^–4^, and 2.8 × 10^–4^). At 96 h p-ea (*p =* 5.9 × 10^–5^), MeJA-300 treatment exhibited the lowest activity among the treatments, significantly lower than the control CW (p = 0.014), followed by α-I-150, although in this latter case, the difference was not statistically significant relative to CW. α-I-300-treated groups showed the highest activity, significantly higher than CW (*p =* 0.034). By 144 h p-ea, significant differences persisted (*p =* 2.6 × 10^–6^), with both α-I-150 and MeJA-300 groups showing lowered P450 activity (*p =* 7.3 × 10^–4^ and 1.1 × 10^–3^). As observed for other enzymes, the MeJA-150-treated group showed the highest activity, significantly greater than that of CW (*p =* 5.9 × 10^–3^).

### Tissue-specific and time-dependent metabolite patterns

To gain a global view of metabolite-based patterns between treatments, initial OPLS-DA analyses were conducted on the entire chemical dataset of leaf and tepal samples. This untargeted metabolite-based profiling revealed substantial differences between leaf and tepal responses and clear treatment‑specific metabolite signatures over time. Quality control confirmed consistent instrument performance throughout the LC–MS analysis, as evidenced by high reproducibility, substantial overlap of the reference QC samples, and tight clustering of the pooled QC samples (Supporting Information, S1 File, Fig S1).

**Fig 3:**
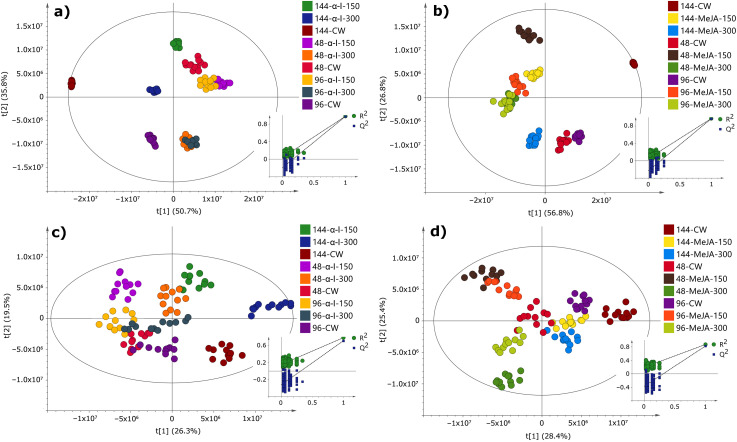
Orthogonal partial least squares discriminant analysis (OPLS-DA) was used to assess metabolite profile variation across treatments in leaf and tepal samples, treated with α-ionone (α-I) and methyl jasmonate (MeJA), and compared to the control (CW), collected at 48, 96, and 144 h post-elicitor application. a) leaf samples after α-I treatment; b) leaf samples after MeJA treatment; c) tepal samples after α-I treatment; d) tepal samples after MeJA treatment. Distinct clustering among treatments reflects differential metabolite-based responses to elicitor application. Each treatment group included six biological and two technical replicates. Each score plot is presented alongside the corresponding permutation plot (200 permutations) (right-bottom plots) per panel to validate the respective OPLS‑DA model, as permuted Q^2^ and R^2^Y distributions were substantially lower than original model values, and permutation regression of the Q^2^ points intersects the vertical axis below zero.

The OPLS-DA analyses revealed apparent differences in metabolite profiles across treatments, tissues, and time points (Fig 3). Specifically, leaf samples displayed pronounced metabolic divergence among treatment groups (Fig 3a–b), while tepal samples showed comparatively weaker separation (Fig 3c–d). In leaf samples, α-I treatment produced a marked separation from the control along Component 1 (C1, 50.7% variance; Fig 3a), with distinct clustering of samples collected at 48, 96, and 144 h p-ea. This pattern indicates that α-I elicited a strong and time-dependent metabolic reprogramming in leaves. Similarly, methyl jasmonate (MeJA) treatment caused a pronounced separation from control samples along C1 (56.8% variance; Fig 3b), suggesting a robust, concentration-dependent metabolic response, particularly evident in the 48- and 144-h samples exposed to 300 ppm MeJA. In contrast, tepal tissues displayed lower explained variance and greater overlap among treatment groups (Fig 3c-d), indicating subtler or more complex metabolic responses. Although some temporal trends were apparent, especially a gradual divergence between early and late sampling points, the separation between concentrations was less evident. In this regard, dual OPLS-DA analyses (Fig S2–S13 in S1 File) confirmed that the metabolite profiles of treated and untreated tepals remained notably different, suggesting that both elicitors induced a weaker metabolic response in tepals than in leaves. These results demonstrate that both elicitors induced tissue-specific, time-dependent changes in the metabolite profiles, with leaves exhibiting stronger and more differentiated responses than tepals, consistent with their greater metabolic plasticity and sensitivity to signaling compounds such as α-I and MeJA.

### Pattern recognition of induced metabolites and their functional classes

As mentioned above, to identify key metabolites induced by the treatments, we performed dual-comparative OPLS-DA analyses of metabolite composition across treatments at each time point for both tepals and leaves. Treatments were selected based on significant phenotypic changes in herbivory and enzyme activity, enabling the identification of metabolites potentially associated with reduced florivory and altered enzyme responses. The following dual comparisons were performed: 1) α-I-300 vs. CW and MeJA-300 vs CW at 48 h post-treatment. 2) α-I-150 vs. CW and MeJA-300 vs. CW at 96 h post-treatment, and 3) α-I-150 vs. CW and MeJA-300 vs. CW at 144 h post-treatment, whose derived score plots are included in the supporting information (S1 File, Fig S2-S13). This binary OPLS-DA-based analysis identified several differential metabolites between the treatments and the control. The identities of these differential metabolites were determined using MS-based annotation, with the results summarized in the supporting information (S2 Table). The metabolite annotations corresponded to level 3, indicating compound-class assignments using tentative candidates rather than confirmed molecular identities, according to the previously proposed identification confidence scale [30].

Based on the comparisons above, the metabolite upregulations relative to the control group were primarily attributed to conjugated flavonoids, with quercetin and kaempferol as the plausible predominant aglycones. Other changes included phenylpropanoids and, to a lesser extent, benzoic acids. In this regard, Table 1 summarizes the number of metabolites that were upregulated in response to α-I-150 and MeJA-300 treatments at three post-treatment intervals (48, 96, and 144 h) in leaf and tepal tissues.

**Table 1:** Summarized metabolite upregulations in tepals and leaves treated with MeJA-300 and α-I-150 during three post-treatment times (48-, 96-, and 144 h).

Metabolite class ^a^	leaves ^b^						tepals ^b^					
	MeJA 48 h	MeJA 96 h	MeJA 144 h	α-I 48 h	α-I 96 h	α-I 144 h	MeJA 48 h	MeJA 96 h	MeJA 144 h	α-I 48 h	α-I 96 h	α-I 144 h
Benzoic acids	0	0	0	0	0	0	2	0	2	2	0	2
Phenylpropanoids	3	4	2	0	3	4	2	2	2	2	2	0
Flavonoids	7	9	12	4	7	10	5	11	9	4	9	7

a Metabolite class according to the annotation confidence level 3. α-I-150 = α-ionone at 150 ppm; MeJA-300 = methyl jasmonate at 300 ppm.

b Data indicate the number of metabolites classified as upregulated based on differential performance (VIP > 1; S3 Table), as identified through OPLS‑DA comparisons between elicitor‑treated samples and the control.

Upregulated metabolite classes were found to be present in both leaves and tepals; however, the chemical composition of these organs differed in both the quantity and identity of specific metabolites. The results revealed clear differences in the metabolic activation patterns between elicitors, tissues, and time points. In leaves, MeJA induced a consistent upregulation of phenylpropanoids and flavonoids across all sampling times, with the highest number of flavonoids (n = 12) detected at 144 h, indicating a progressive stimulation of phenylpropanoid-derived secondary metabolism. Application of α-I also promoted flavonoid accumulation, particularly at 144 h, when ten metabolites were upregulated, but its effects on benzoic acids were comparatively modest. In tepals, MeJA treatment led to the upregulation of mainly flavonoids, with the most pronounced induction observed at 96 and 144 h. Plants treated with α-I showed a similar trend, though with slightly fewer upregulated compounds, suggesting a weaker or more transient response. Remarkably, benzoic acid derivatives were only upregulated in tepals and not in leaves, indicating a tissue-specific metabolic response possibly associated with floral scent or pigment biosynthesis.

This observation was further supported by examination of individual metabolite patterns summarized in supporting information (S3 Table and S4 File). In leaves, MeJA treatment caused a sustained upregulation of several phenylpropanoids (including feruloylquinic, sinapoylshikimic, and caffeoyltartaric acids) as well as multiple flavonoids such as kaempferol, myricetin, and kaempferol mono- and triglycosides, revealing an early and persistent activation of the phenylpropanoid–flavonoid pathway. Elicitation by α-I also induced upregulation of related metabolites, notably quercetin derivatives and kaempferol conjugates. However, its effects were occasionally accompanied by transient downregulation events (e.g., for sinapic and cinnamoylglucoside acids), indicating a more modulated metabolic response. In tepals, MeJA treatment produced a pronounced upregulation of caffeoylshikimic acid, syringoylmalic acids, and several glycosylated flavonoids, including quercetin glucoside glucuronide, tetramethylquercetin rutinoside, and acacetin derivatives, suggesting active remodeling of secondary metabolism. Conversely, α-I treatment resulted in both up- and downregulation within the same metabolite classes, particularly for quercetin and kaempferol conjugates, suggesting temporally dynamic adjustments in metabolic fluxes. Benzoic acid derivatives were slightly upregulated in tepals rather than leaves, confirming tissue-specific specialization in metabolite biosynthesis.

### Integration of Florivory, Enzyme, and Metabolite-Based Patterns

The combined dataset indicates that MeJA‑300 and α-I‑150 provide the strongest defensive outcomes. Treatments that reduced florivory also corresponded to the most pronounced reductions in GST or P450 activity and the strongest shifts in metabolite profiles. While metabolite identities remain tentative (level 3), chemical class‑level trends strongly support elicitor‑induced activation of secondary metabolism, likely contributing to altered thrips detoxification capacity. In addition, although correlations between metabolite enrichment and enzyme activity were observed, establishing causality requires targeted assays using purified compounds or fractionated extracts.

## Discussion

### Overview and Contextualization of Key Findings

This study demonstrates that exogenous application of MeJA and α-I reduces florivory caused by WFT in *Alstroemeria*. The efficacy of elicitor treatments in reducing florivory exhibited marked temporal dependence. MeJA-300 significantly suppressed florivory at 48 h post-elicitation; however, this protective effect progressively attenuated thereafter. In contrast, α-I-treated plants displayed a delayed reduction in florivory, with the response becoming evident at 144 h post-elicitation. MeJA induced a rapid but transient defense response, consistent with its role as a direct activator of the jasmonate signaling cascade, in which defense gene expression peaks in the post-elicitation period [32] and subsequently attenuates due to feedback suppression mediated by JAZ repressor proteins [33,34]. In contrast, α-ionone could elicit a delayed and more sustained protective response, acting through a JA-independent pathway that upregulates β-1,3-glucanase and chitinase genes [18]. This mechanistic distinction suggests that MeJA and α-ionone engage fundamentally different regulatory routes: the former triggers a fast-acting but self-limiting hormonal response, and the latter activates a slower but potentially more durable defense state more consistent with a priming-type mechanism. The protective effect of MeJA observed in the present study is consistent with previous reports in vegetable and model systems, where MeJA enhances resistance to thrips and aphids via jasmonate-dependent signaling and the induction of defense-related metabolites [19,35,36]. Evidence in ornamental crops remains limited, and our findings extend elicitor-based defense strategies to floriculture systems.

Moreover, the reduction of key detoxification enzymes (e.g., GST, EST, and P450) in thrips feeding on elicitor-treated plants suggests that elicitor-induced metabolic shifts in *Alstroemeria* plants might disrupt the pest’s detoxification machinery and capacity. α-IIntegrating florivory, enzymatic, and metabolite profile data reveals that both elicitors might activate plant-derived chemical pathways associated with antifeedant effects, albeit with distinct temporal dynamics and magnitudes [37]. Specifically, the most effective treatments (i.e., MeJA-300 at 48 h p-ea and α-I-150 at 144 h p-ea α-I) elicited the greatest reductions in feeding, alongside the most significant modulation of detoxification enzymes. These results suggest elicitor- and time-specific influences on plant-herbivore interactions, whose causal relationships warrant exploration in future experiments. Furthermore, the correlation between reduced florivory and impaired detoxification highlights elicitor-based strategies as viable, sustainable alternatives to synthetic insecticides [38,39].

### Plant responses to elicitors affecting insect feeding

Exogenous application of signaling mimics such as jasmonates and apocarotenoids can prime plant defense mechanisms and represents a promising strategy for triggering plant immunity [18,40,41], leading to faster and stronger inducible responses, even before an herbivore attack, activating a wide range of metabolic pathways that lead to the synthesis of specialized metabolites that affect herbivores either directly or indirectly [13,42]. Consequently, herbivores exposed to such responses may exhibit reduced feeding, suppressed detoxification, impaired digestion, reduced oviposition, or increased mortality [18,42,43]. In the present study, we observed that *Alstroemeria* plants responded to α-I and MeJA elicitation by activating metabolite-related defenses in tepals, thereby significantly reducing the florivory rate of WFT. Tepals are physiologically distinct from vegetative tissues, exhibiting high metabolic activity, intense pigmentation, and accumulation of secondary metabolites associated with both defense and floral signaling [44]. Their exposure, nutrient content, and surface characteristics make them especially susceptible to florivorous thrips [45]. Consequently, assessing thrips feeding directly on tepals provides a biologically relevant and commercially meaningful measure of plant resistance, allowing evaluation of defensive responses induced by elicitor treatments in the tissue most directly linked to economic losses in *Alstroemeria* production [2]. Focusing on tepals, therefore, ensures that observed antifeedant effects are immediately interpretable in the context of floriculture quality and pest management outcomes.

The main success of this strategy ultimately relies on the ecological and physiological consequences that induced defense provokes in herbivorous insect populations. The activation of metabolite-related responses can reduce feeding through deterrence [46], impair development by slowing the weight-gain rate [52], deter oviposition [18], and even alter enzymatic dynamics in herbivorous insects [47], among other effects. Consistent with these reports, elicitation in *Alstroemeria* appeared to diminish WFT detoxification functions, likely contributing to reduced feeding.

### Enzyme activity decreased, and antifeedant effects were induced by ingestion of elicited tepals

Insects depend upon GSTs, P450s, and ESTs to metabolize plant-derived xenobiotics and insecticides [5,47]. Suppression of these systems compromises resistance and feeding performance [48]. Therefore, the reduction of detoxification activity by plant metabolites is a primary mechanism [15] through which induced plant defenses bolster protection against herbivores. In our study, reduced enzyme activity was observed concurrent with decreased florivory (Fig 2), suggesting a plausible association. Under this context, three non-exclusive explanations may account for this pattern. First, potential enzyme inhibition due to flavonoid action, followed by a posterior antifeedant effect. This rationale is consistent with previous findings, as flavonoids are known to inhibit detoxifying enzymes, particularly GST and P450 [49,50]. Such inhibition should increase adverse effects on herbivore physiology by reducing their ability to metabolize other ingested harmful xenobiotics, thereby enhancing plant defenses and producing direct antifeedant effects. In addition, flavonoids can promote antifeedant responses, including starvation-mediated effects [51], and disrupt oxidative balance or modulate host physiology through altered signaling pathways, gene expression, and post-translational modifications [15]. Therefore, the consistent reduction in enzyme activity across treatments, particularly in MeJA-300 at 48 h p-ea and α-I-150 at 144 h p-ea, suggests a plausible association between metabolite induction and decreased thrips tolerance, warranting further exploration in future studies.

A second possibility involves a rapid, direct sublethal effect of the elicited metabolites on the insect, occurring before the detoxification enzyme-based response is fully activated. This alternative could explain the observed reduction in enzyme activity. Some studies have reported that sublethal exposure to insecticidal compounds can decrease the activity of detoxifying enzymes, such as GST and carboxylesterases (CarE) [52], suggesting that elicited metabolites may interfere with detoxification pathways prior to full enzymatic induction. Consequently, the reduced enzymatic responses observed in thrips might reflect starvation, metabolic stress, and/or compensatory modulation rather than direct chemical suppression alone.

A third and consequential possibility is a non-adaptive response, indicating that the thrips’ detoxification system was not equipped to handle the elicited metabolites, ultimately leading to detoxification failure. Although thrips are cosmopolitan and generalist herbivores capable of feeding on more than 200 plant species [53], they may still be vulnerable to particular compounds produced through elicitor-induced pathways. If the plant response involves unique or uncommon metabolites, this could outpace the thrips’ enzyme adaptations, resulting in suboptimal or ineffective detoxification. A previous study demonstrated that generalist insects do not necessarily possess broader detoxification repertoires than specialists [54]. These insights align with our findings, suggesting that the specific metabolites elicited by α-I and MeJA may exceed the thrips’ responsive enzymatic capacity, leading to system overload and directly contributing to the observed reductions in enzyme activity and florivory.

GST and P450 activity reductions were consistent across time points, whereas the EST activity decreased primarily at 96–144 h. This outcome is particularly important because GST and P450 increase the hydrophilicity of xenobiotics through conjugation and hydroxylation [55,56], thereby facilitating excretion. Although limited information exists on the specific bioactivity of several flavonol derivatives annotated in this study, their structural similarity to known enzyme inhibitors, such as taxifolin and quercetin, suggests potential relevance to plant–insect chemical interactions. In this regard, flavonoids, particularly those identified in conifer species, have demonstrated potent *in vitro* inhibition of detoxification enzymes, including GST and esterases, which are central to insecticide resistance mechanisms [57,58]. Similarly, flavonoid-rich extracts from *Pinus* and *Abies* species exhibited GST inhibition comparable to or even superior to that of known inhibitors such as diethyl maleate [59]. Given the crucial roles of GSTs and cytochrome P450 monooxygenases in xenobiotic detoxification and oxidative stress tolerance [60], their inhibition by flavonoids may compromise herbivore defense systems, increasing susceptibility to both endogenous plant allelochemicals and applied insecticides.

Regarding esterases, these enzymes typically interact with ester bonds, helping break down certain xenobiotics and contributing to their metabolism and excretion. While they are not directly involved in processing glycosylated molecules [61], they may interact with some esterified forms (e.g., acetylated or malonylated) of glycosylated flavonoids found in our study. Additionally, some studies have shown that certain carbohydrate esterases can act on glycosylated flavonoids, suggesting potential interactions between these enzymes and glycosylated xenobiotics [62]. However, the observed reduction in esterase activity might represent a secondary effect resulting from the influence on GST and P450 enzymes, which likely triggered strong antifeedant responses and, consequently, reduced feeding and overall metabolic activity in the insects, including esterase activity.

Given the rationale outlined above, analyzing the metabolic profiles of herbivore excretions from individuals fed on elicitor-treated plants would be highly valuable. This approach may provide insights into shifts in xenobiotic metabolism and reveal the specific metabolites processed by the gut enzyme pool. With this strategy, it would be possible to infer the types of enzymatic reactions occurring in the insect gut, such as hydrolysis, oxidation, or conjugation with molecules like glutathione or sugars, to facilitate excretion. This information would not only clarify how insects process plant-derived xenobiotics but also help identify potential biochemical bottlenecks in which elicitor-induced metabolites can overwhelm or impair detoxification pathways. Moreover, understanding the precise molecular and cellular mechanisms underlying these reactions could provide a more integrative view of how elicitation affects herbivores and, in turn, guide the development of synergistic management strategies that combine elicitors with other control approaches.

### Metabolite-related response of tepals and leaves to MeJA and α-I elicitation

The observed patterns, consistent with the PLS-DA comparisons, revealed that most of the top-ranked features (based on VIP scores > 1) were glycosylated flavonoids, particularly quercetin and kaempferol derivatives. These upregulated metabolites detected in tepals and leaves suggested that elicitor treatments primarily trigger the accumulation of flavonoids, with minor contributions from other phenolic compound classes, such as phenylpropanoids (Table 1). The combined metabolite-based evidence demonstrates that MeJA is a strong and consistent elicitor of phenylpropanoid and flavonoid biosynthesis in both leaves and tepals. In contrast, α-I induces a more variable, concentration- and time-dependent modulation. The robust induction of phenylpropanoid and flavonoid compounds under MeJA treatment is consistent with its established role as a signaling molecule that activates jasmonate-dependent defense pathways and secondary metabolite accumulation [63]. On the other hand, α-I appears to trigger more selective or transient responses, potentially acting through distinct signaling routes or modulating specific branches of the same biosynthetic network [64]. Both elicitors induce temporally dynamic regulation of specialized metabolism, characterized by a short-term, waning effect from MeJA and a characteristically delayed response from α-I. In this context, the metabolite profiles of leaves and flowers exhibited marked contrasts in their regulatory responses, reflecting their divergent physiological roles. Notably, anthocyanins in the tepals remained largely unaffected by either elicitor, maintaining consistently high levels. Given that flowers function as strong metabolic sinks and play a direct role in plant reproductive fitness, they are thought to rely primarily on constitutive rather than inducible defense mechanisms [65]. Nonetheless, multiple interconnected factors, such as tissue-specific metabolic capacity, differential elicitor perception, and resource allocation priorities, may collectively contribute to the distinct metabolite responses observed between floral and foliar tissues.

First, many metabolites synthesized in the leaves may be translocated to the tepals to reinforce chemical defense in reproductive organs. For instance, in *Lupinus albus* (Magnoliopsida: Fabaceae), quinolizidine alkaloids are produced in the leaves but transported to various plant tissues, including flowers, where they tend to accumulate [66]. In our particular case of flavonoid upregulation, several studies suggest that flavonoids synthesized in vegetative organs may be translocated to reproductive tissues. For instance, plant GSTs have been proposed to act as carrier proteins mediating vacuolar sequestration and long-distance transport of flavonoids, highlighting their key role in regulating tissue-specific accumulation [67]. Other enzymes have also been associated with this long-distance transport role, including MATE (Multidrug And Toxic compound Extrusion) transporters and BTL-like (bilitranslocase like) proteins, which are thought to facilitate flavonoid export or phloem loading for delivery to distal tissues such as petals and seeds [68,69]. This translocation mechanism plays an ecological role, as flavonoids and other translocated metabolites are known to contribute to systemic chemical defenses against herbivores [70,71].

Second, tepals are typically associated with pigment accumulation because of their visual role in pollinator attraction, particularly through the presence of anthocyanins. However, there is evidence that anthocyanins play essential roles in plant defense mechanisms [72] beyond their role in coloration. While no upregulated anthocyanins were detected in tepal samples of treated plants, the anthocyanin-related precursors (e.g., flavonols) were clearly upregulated in leaves and even in tepals. This pattern could indicate tissue-specific regulation of flavonol biosynthesis. Although some flavonoids are known to be translocated between organs [73], flavonol accumulation is also believed to result primarily from local synthesis. This idea is further supported by evidence that flavonol biosynthesis is tightly regulated at the transcriptional level by tissue-specific transcription factors, which control the expression of key biosynthetic genes [74]. According to the above observations, while certain specialized metabolites are synthesized locally within floral tissues, others are either absent or derived from precursors translocated from metabolically active source tissues, such as leaves. This differential metabolic partitioning between plant organs likely contributes to the marked divergence in defense-related regulatory responses recognized between leaves and tepals, reflecting the functional specialization and distinct metabolic capacities of these tissues.

### Elicitor-Induced Metabolic Reprogramming and Agroecological Implications

The exogenous application of MeJA-300 at 48 h p-ea or α-I-150 at 144 h p-ea effectively alters the metabolic profile of *Alstroemeria* leaves and tepals, leading to a significant reduction in thrips feeding rates on floral tissues. In an agroecosystem context, this effect could be particularly advantageous, consistent with α-I effects reported in tomato, including reductions in thrips survival and oviposition rates in tomatoes without direct insecticidal activity after elicitor treatment, thereby constraining population growth [18]. Application of α-I also reduced survival and larval weight in *Spodoptera litura* [18], suggesting broader activity against noctuids, which are relevant to *Alstroemeria* production. Specifically, MeJA substantially reshapes plant metabolomes [17] and can recruit natural enemies [19,75]. In the present study, MeJA-300 induced an early defense peak that declined over time, consistent with a two-stage jasmonate response [76]. The α-I-150 treatment, in contrast, exhibited prolonged effects, possibly due to jasmonate-independent gene activation [18], although this hypothesis requires further study.

Flavonoid-mediated inhibition of detoxification enzymes may be particularly valuable against resistant thrips populations [6,77] and may synergize with insecticides [49,50]. Elicitor-induced metabolic priming represents a strategic advancement for integrated pest management (IPM). By interacting with insecticides, these elicitors could enhance efficacy and reduce the need for higher dosages or more frequent rotations of active ingredients. However, the compatibility of these treatments with specific insecticides and their impact on agroecosystem biodiversity requires rigorous empirical validation.

Although the present study did not evaluate volatile emissions, it is worth noting that exogenous application of MeJA artificially activates defense pathways that are also engaged under natural biotic stress conditions [78]. Jasmonic acid (JA)-dependent signaling is well-documented as a primary response to herbivory and pathogen attack, and while the JA-independent pathway activated by apocarotenoids such as α-ionone is less characterized under field conditions, it similarly culminates in the induction of plant defense [18]. In ornamental crops such as *chrysanthemum*, feeding by WFT has been documented to alter the plant VOC blend, increasing the emission of Germacrene-D and β-caryophyllene, in ways that attract the predatory mite *Neoseiulus cucumeris* [79]. Furthermore, thrips-induced VOC responses in tomato have been demonstrated to be JA-dependent; for instance, wild-type plants significantly increased terpene production in response to WFT feeding, while JA-deficient *def-1* mutants did not, and exogenous MeJA application significantly increased terpene-based volatile production in both wild-type and JA-deficient plants, directly linking jasmonate signaling to VOC-mediated defense against WFT [80]. The extent to which the defense activation documented in the present study leads to the production of analogous volatile compounds in *Alstroemeria*, and whether such signals may mediate interactions with natural enemies of WFT, remain important questions that warrant further investigation.

In addition, while the results are promising, several actions must be taken before translating these findings into practical applications. Although the elicitation treatments successfully induced the production of defensive compounds in the plant, it is essential to evaluate the persistence of this effect throughout the entire crop cycle. Such an evaluation is necessary to determine the specific protection window provided. These findings, though significant under controlled, Petri dish-based conditions, warrant validation using whole inflorescences to account for potential artifacts from tepal excision. Therefore, field-level trials are required to assess the persistence of elicited metabolite-based defenses, their systemic movement in commercial production environments, and their broader ecological interactions [81]. These actions include optimizing elicitor concentration, timing, and application methods, as well as evaluating potential trade-offs between defense activation and key horticultural traits such as plant health, flowering longevity, and marketability in *Alstroemeria*. Additionally, although untargeted metabolomics identified several candidate metabolites and compound classes, these were assigned with level 3 confidence. Targeted validation using authentic standards and functional assays (e.g., RNAi knockdown of GST in thrips) will be necessary to confirm compound identities and clarify mechanistic links. Parallel assessments of non-target impacts on beneficial arthropods and overall agroecosystem health will help ensure ecological compatibility. Finally, expanding efficacy trials to other economically essential pests will help determine the broader applicability of α-I and MeJA in sustainable pest management strategies for ornamental and agricultural systems. These implications do not undermine the central findings but highlight areas for further investigation to strengthen mechanistic interpretation and practical application.

## Conclusions

Exogenous application of α‑ionone and methyl jasmonate effectively enhances chemical defenses in *Alstroemeria* and reduces WFT feeding. The treatments MeJA‑300 and α-I‑150 produced the most robust antifeedant effects and elicited substantial changes in metabolite profiles, thereby altering detoxification enzyme activity. The induction of flavonoid- and phenylpropanoid-associated features provides a biochemical context for the observed reductions in florivory and enzyme activity modulation. These results support the potential of α-I and MeJA as components of sustainable thrips management strategies in ornamental crops. Future studies should focus on validating metabolite identities, clarifying causal links between specific compounds and enzyme inhibition, and assessing field-level performance, persistence, and integration with existing pest management programs.

## Supporting information

S1 File Additional OPLS-DA-derived score plots. (PDF)

S1 Table Compilation of annotated metabolites. (PDF)

S2 Table Overview of up-/downregulated, annotated metabolites. (PDF)

S2 File Data of OPLS-DA models. (PDF)
