# Supplementary material for: Exogenous application of α-Ionone and Methyl jasmonate induces metabolite-based response in Alstroemeria and reduces feeding damage by Western Flower Thrips
Source: PLoS One. 2026 Aug 11;21(8):e0355314. doi: 10.1371/journal.pone.0355314 (PMC13460579; doi:10.1371/journal.pone.0355314)
Supplement: Supplementary file 1 [file pone.0355314.s001.pdf]

S1 File
(PDF)

Supplementary OPLS-DA-derived plots (Fig S1-S13)

Content

		**Pag**
**Fig S1.**	Partial Least Squares Discriminant Analysis (OPLS-DA) for metabolite profile variation across treatments in leaf (A) and tepal (B) samples	**2**
**Fig S2.**	OPLS-DA score plot showing the dual comparison of metabolic profiles between control (CW) and α-I-150 treated samples at 48 hours post-treatment.	**3**
**Fig S3.**	OPLS-DA score plot showing the dual comparison of metabolic profiles between control (CW) and MeJA-300 treated samples at 48 hours post-treatment.	**3**
**Fig S4.**	OPLS-DA score plot showing the dual comparison of metabolic profiles between control (CW) and α-I-150 treated samples at 96 hours post-treatment.	**4**
**Fig S5.**	OPLS-DA score plot showing the dual comparison of metabolic profiles between control (CW) and MeJA-300 treated samples at 96 hours post-treatment.	**4**
**Fig S6.**	OPLS-DA score plot showing the dual comparison of metabolic profiles between control (CW) and α-I-150 treated samples at 144 hours post-treatment.	**5**
**Fig S7.**	OPLS-DA score plot showing the dual comparison of metabolic profiles between control (CW) and MeJA-300 treated samples at 144 hours post-treatment.	**5**
**Fig S8.**	OPLS-DA score plot showing the dual comparison of metabolic profiles between control (CW) and α-I-150 treated samples at 48 hours post-treatment.	**6**
**Fig S9.**	OPLS-DA score plot showing the dual comparison of metabolic profiles between control (CW) and MeJA-300 treated samples at 48 hours post-treatment.	**6**
**Fig S10.**	OPLS-DA score plot showing the dual comparison of metabolic profiles between control (CW) and α-I-150 treated samples at 96 hours post-treatment.	**7**
**Fig S11.**	OPLS-DA score plot showing the dual comparison of metabolic profiles between control (CW) and MeJA-300 treated samples at 96 hours post-treatment.	**7**
**Fig S12.**	OPLS-DA score plot showing the dual comparison of metabolic profiles between control (CW) and α-I-150 treated samples at 144 hours post-treatment.	**8**
**Fig S13.**	OPLS-DA score plot showing the dual comparison of metabolic profiles between control (CW) and MeJA-300 treated samples at 144 hours post-treatment.	**8**

Global OPLS-DA of leaf (A) and tepal (B) samples with Quality Controls (Reference and pooled):

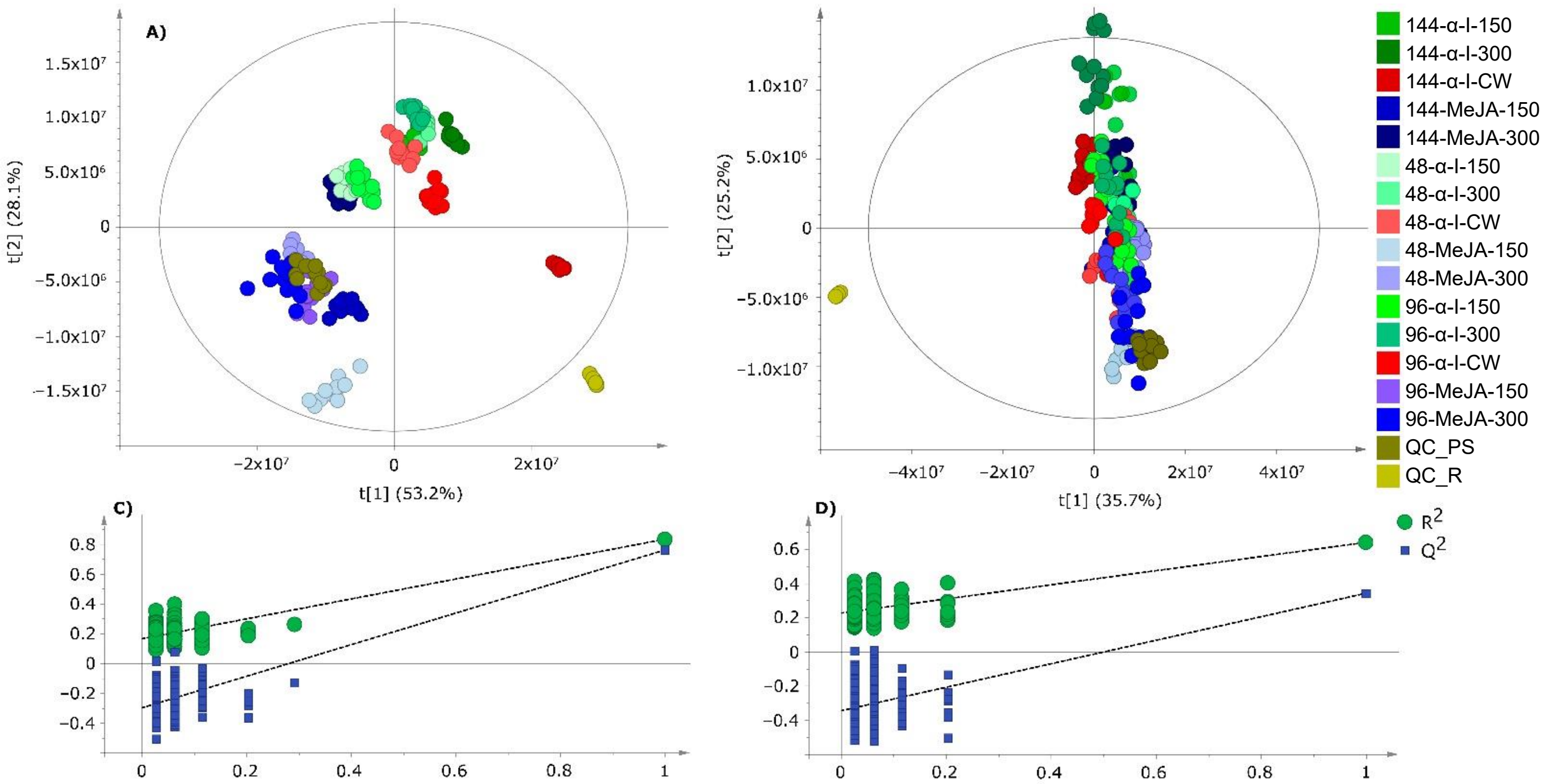

Fig S1. Orthogonal Partial Least Squares Discriminant Analysis (OPLS-DA) was performed to evaluate metabolite profile variation across treatments with methyl jasmonate (MeJA) and α-ionone (α-I) at 300 and 150 ppm in leaf (**A**) and tepal (**B**) samples, across 144, 96, and 48 h post-elicitation. Distinct treatment clusters reflect differences in metabolomic responses to the elicitors. Each treatment group includes four biological replicates and three technical replicates (n = 12). Quality control samples, QCR (reference) and QCPS (pool sample), were included with twelve replicates along the analysis sequence. QCR replicates appeared to overlap tightly, while QCPS replicates formed compact clusters, indicating high reproducibility and analytical robustness of the LC-MS data acquisition. Bottom panels represent the permutation test (n = 200) validating the OPLS-DA model in leaf (**C**) and tepal (**D**) samples. Permuted Q^2 and R^2Y distributions were substantially lower than original model values, and permutation regression of the Q^2 points intersects the vertical axis below zero, confirming the absence of overfitting.

Leaf dual OPLS-DA compared to untreated samples (CW = Control):

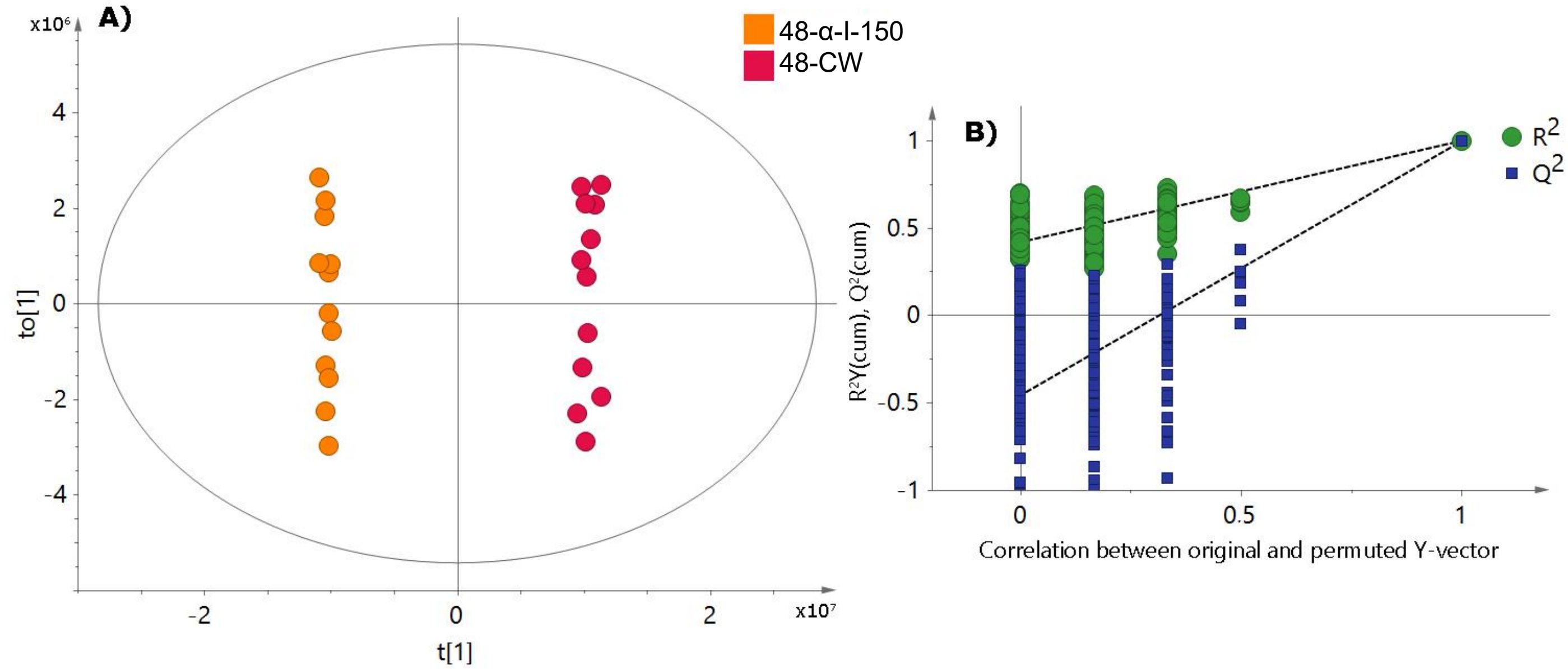

Fig S2. A) OPLS-DA-derived score plot showing the dual comparison of metabolic profiles between control (CW) and α-I-150 treated samples at 48 hours post-treatment. **B)** Permutation test (n = 200) validating the OPLS-DA model. Permuted Q^2 and R^2Y distributions were substantially lower than original model values, and permutation regression of the Q^2 points intersects the vertical axis below zero, confirming the absence of overfitting.

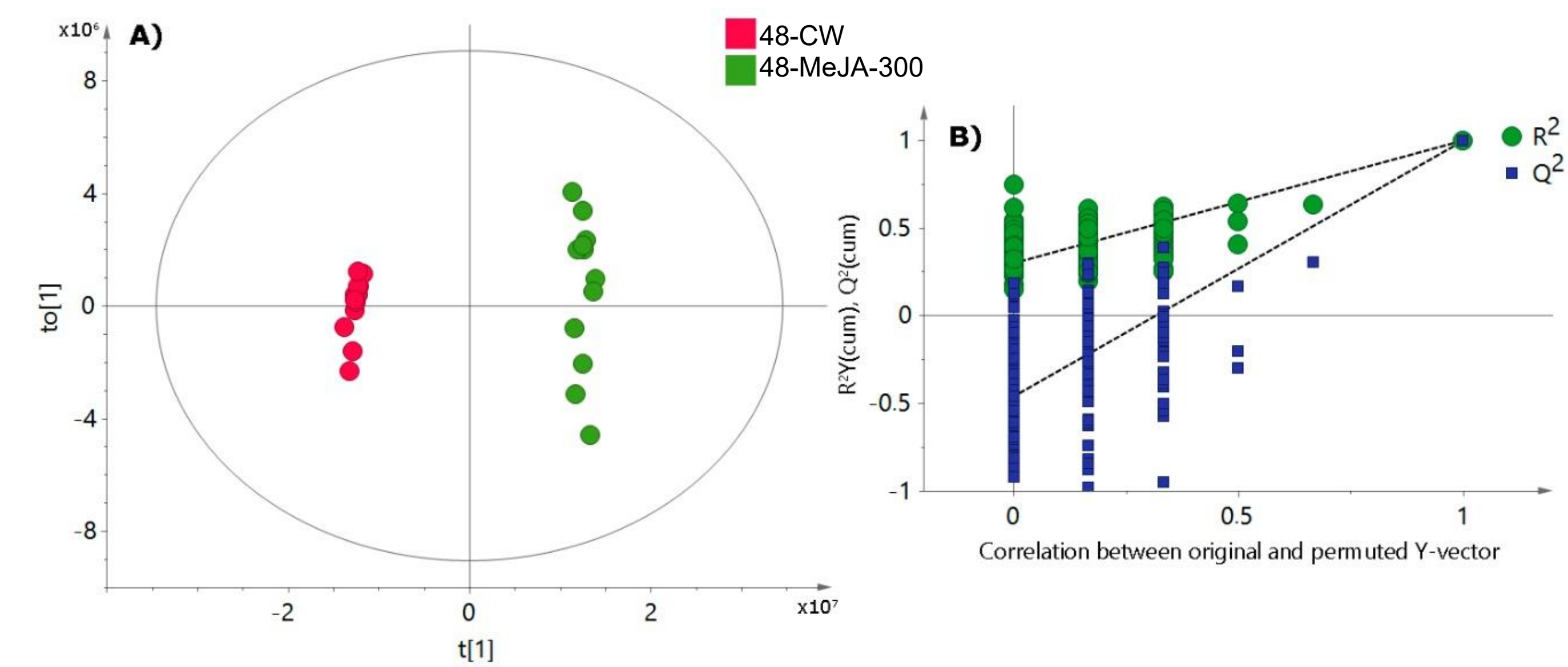

Fig S3. A) OPLS-DA-derived score plot showing the dual comparison of metabolic profiles between control (CW) and MeJA-300 treated samples at 48 hours post-treatment. **B)** Permutation test (n = 200) validating the OPLS-DA model. Permuted Q^2 and R^2Y distributions were substantially lower than original model values, and permutation regression of the Q^2 points intersects the vertical axis below zero, confirming the absence of overfitting.

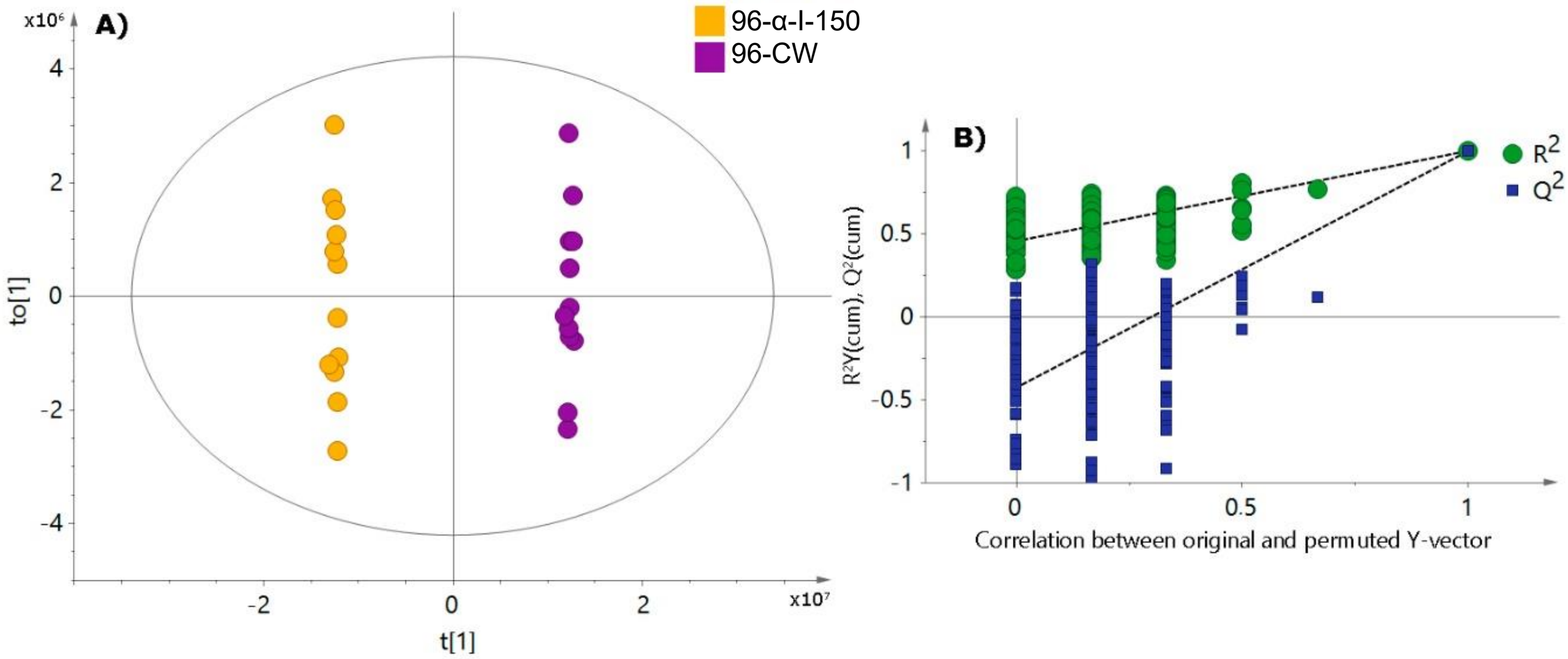

Fig S4. A) OPLS-DA-derived score plot showing the dual comparison of metabolic profiles between control (CW) and α-I-150 treated samples at 96 hours post-treatment. **B)** Permutation test (n = 200) validating the OPLS-DA model. Permuted Q^2 and R^2Y distributions were substantially lower than original model values, and permutation regression of the Q^2 points intersects the vertical axis below zero, confirming the absence of overfitting.

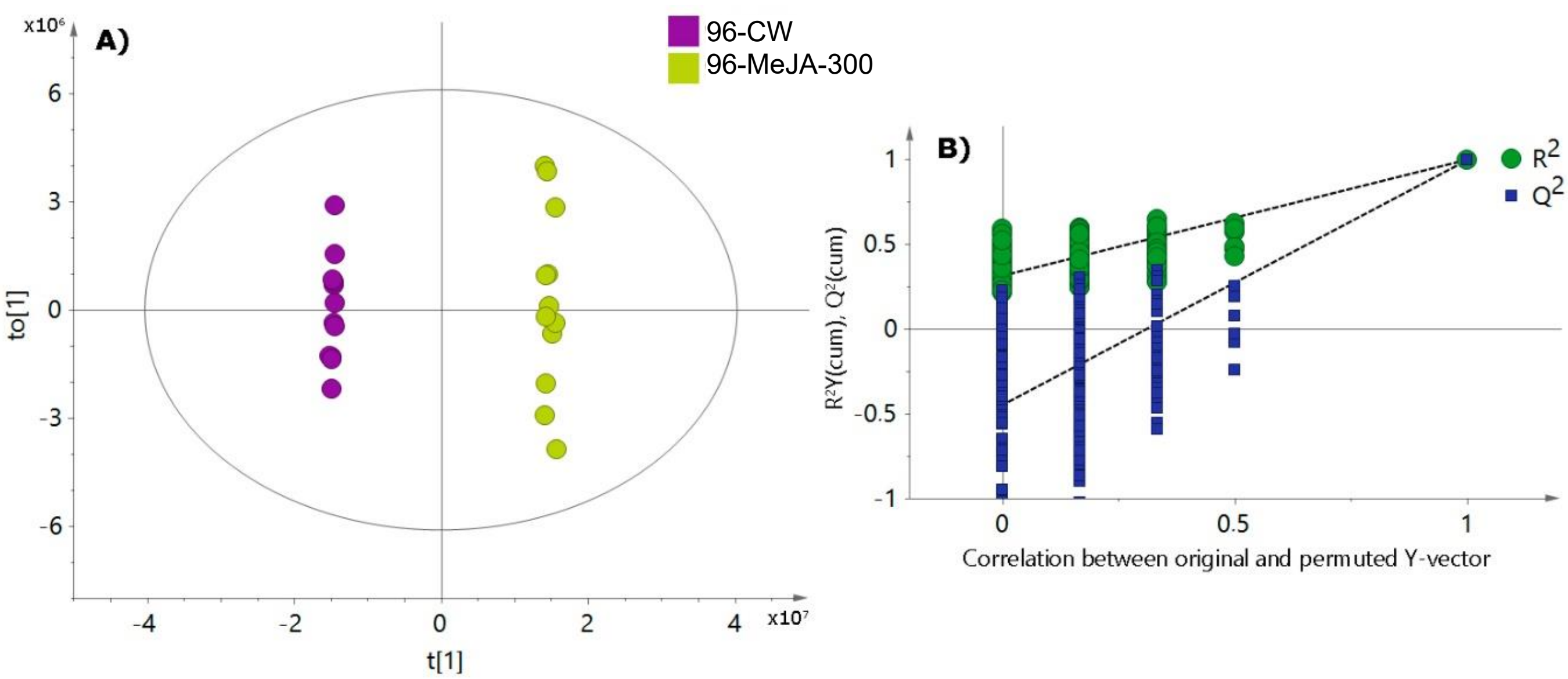

Fig S5. A) OPLS-DA-derived score plot showing the dual comparison of metabolic profiles between control (CW) and MeJA-300 treated samples at 96 hours post-treatment. **B)** Permutation test (n = 200) validating the OPLS-DA model. Permuted Q^2 and R^2Y distributions were substantially lower than original model values, and permutation regression of the Q^2 points intersects the vertical axis below zero, confirming the absence of overfitting.

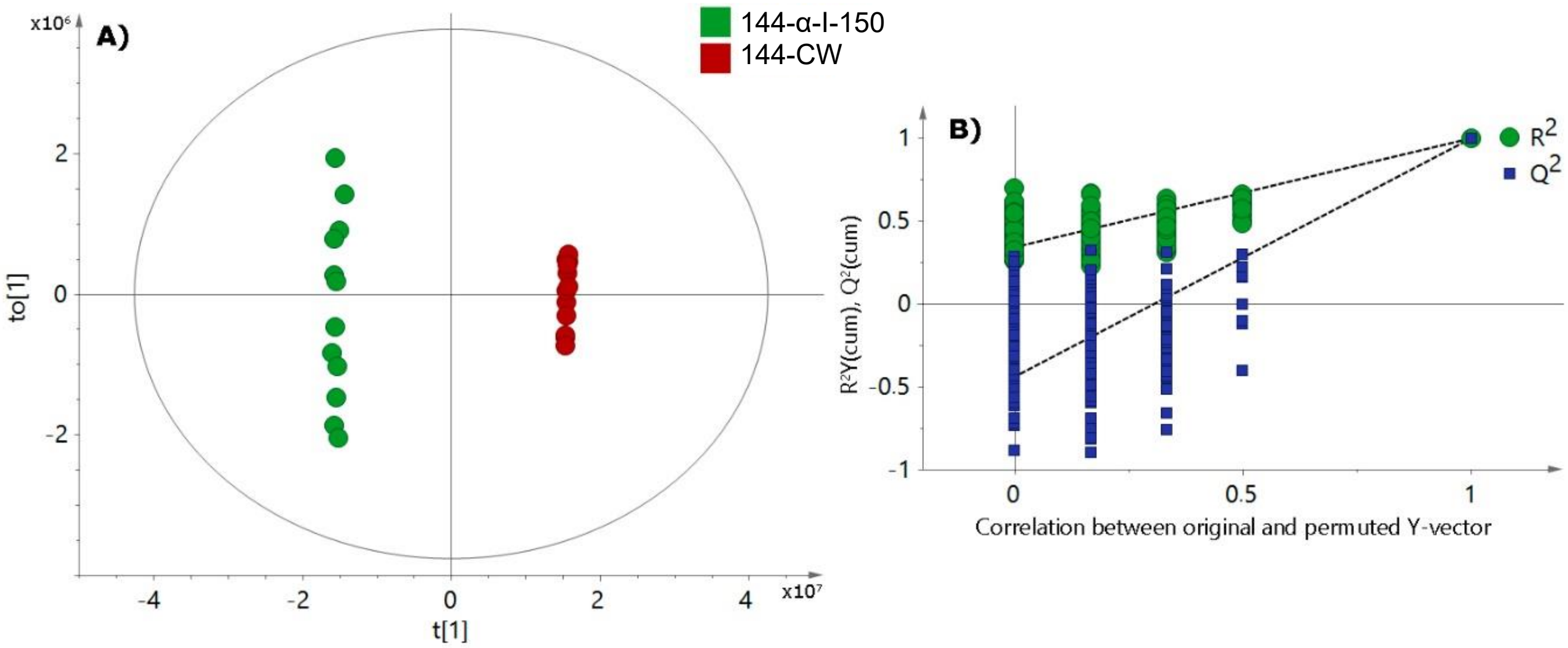

Fig S6. A) OPLS-DA-derived score plot showing the dual comparison of metabolic profiles between control (CW) and α-I-150 treated samples at 144 hours post-treatment. **B)** Permutation test (n = 200) validating the OPLS-DA model. Permuted Q^2 and R^2Y distributions were substantially lower than original model values, and permutation regression of the Q^2 points intersects the vertical axis below zero, confirming the absence of overfitting.

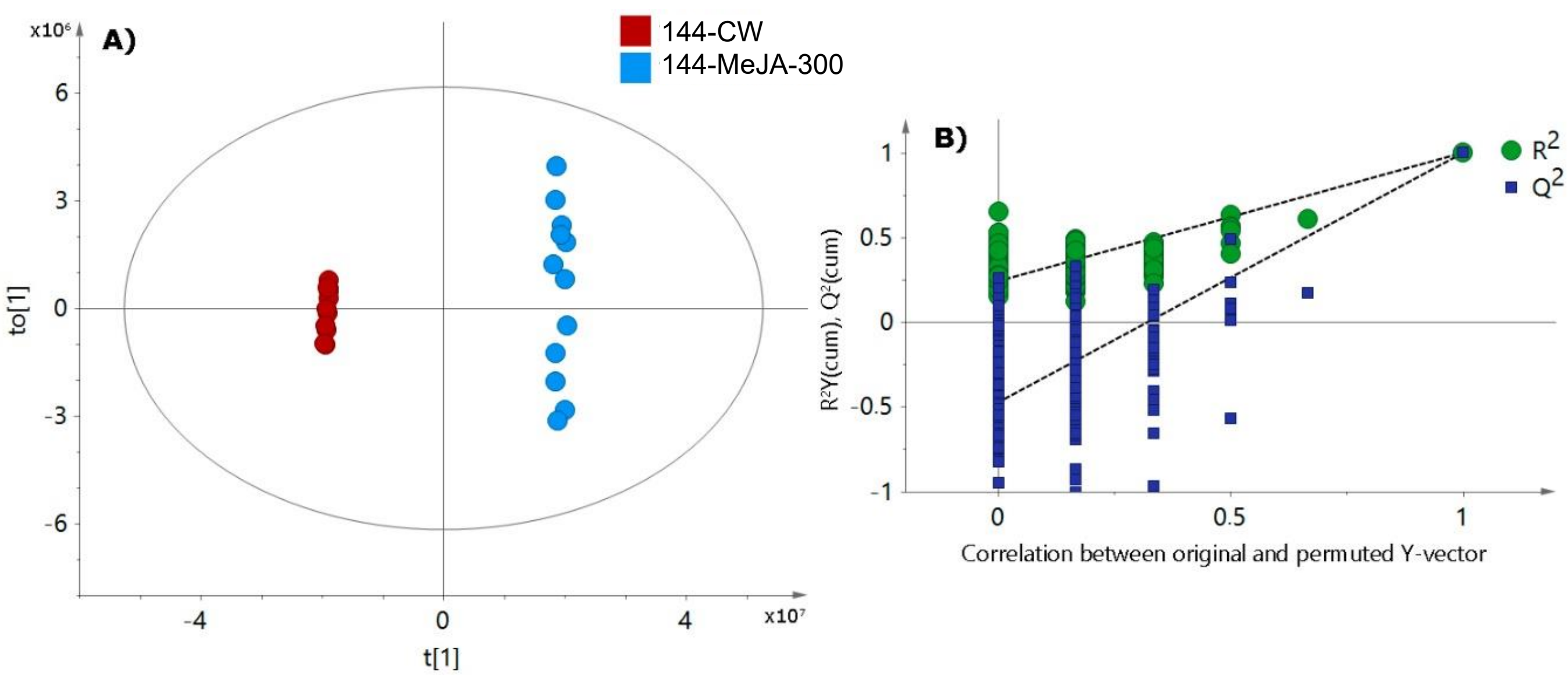

Fig S7. A) OPLS-DA-derived score plot showing the dual comparison of metabolic profiles between control (CW) and MeJA-300 treated samples at 144 hours post-treatment. **B)** Permutation test (n = 200) validating the OPLS-DA model. Permuted Q^2 and R^2Y distributions were substantially lower than original model values, and permutation regression of the Q^2 points intersects the vertical axis below zero, confirming the absence of overfitting.

<u>Tepal</u> dual OPLS-DA compared to untreated samples (CW = Control):

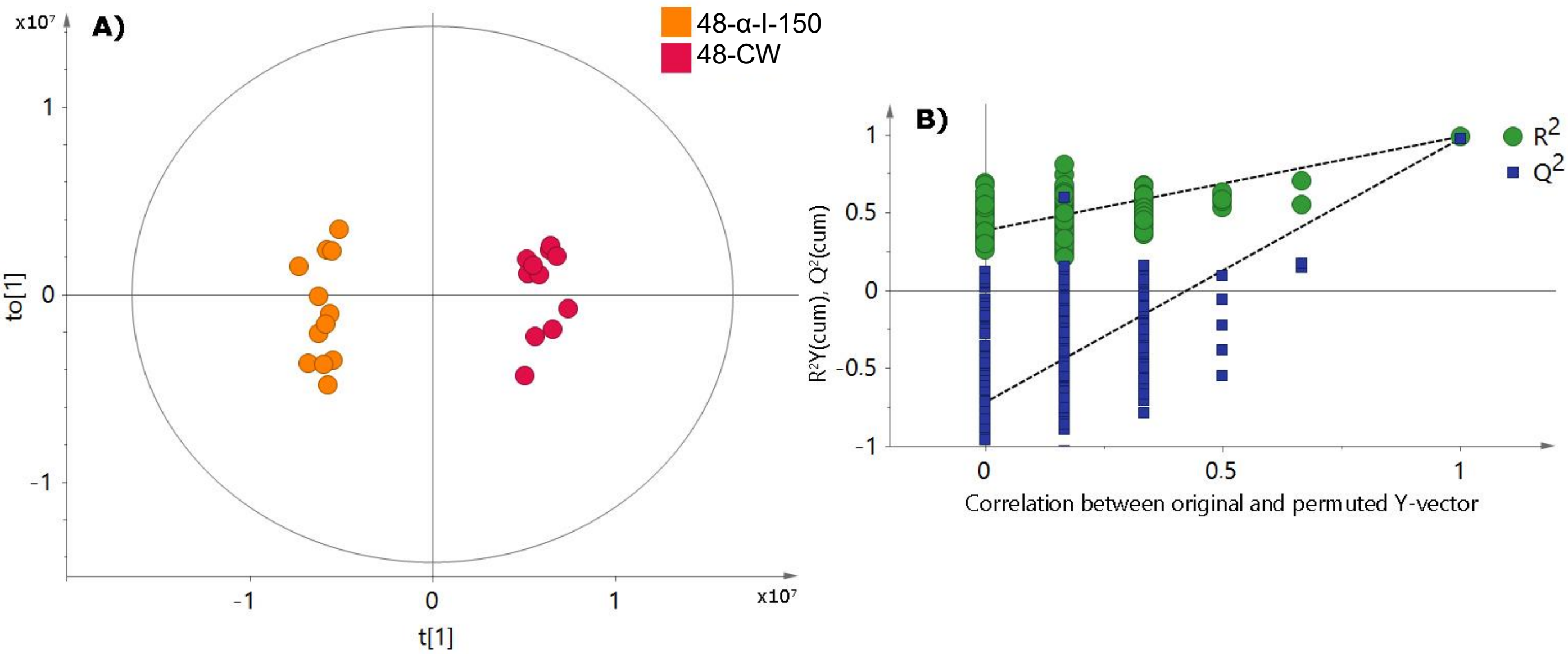

Fig S8. A) OPLS-DA-derived score plot showing the dual comparison of metabolic profiles between control (CW) and α-I-150 treated samples at 48 hours post-treatment. **B)** Permutation test (n = 200) validating the OPLS-DA model. Permuted Q^2 and R^2Y distributions were substantially lower than original model values, and permutation regression of the Q^2 points intersects the vertical axis below zero, confirming the absence of overfitting.

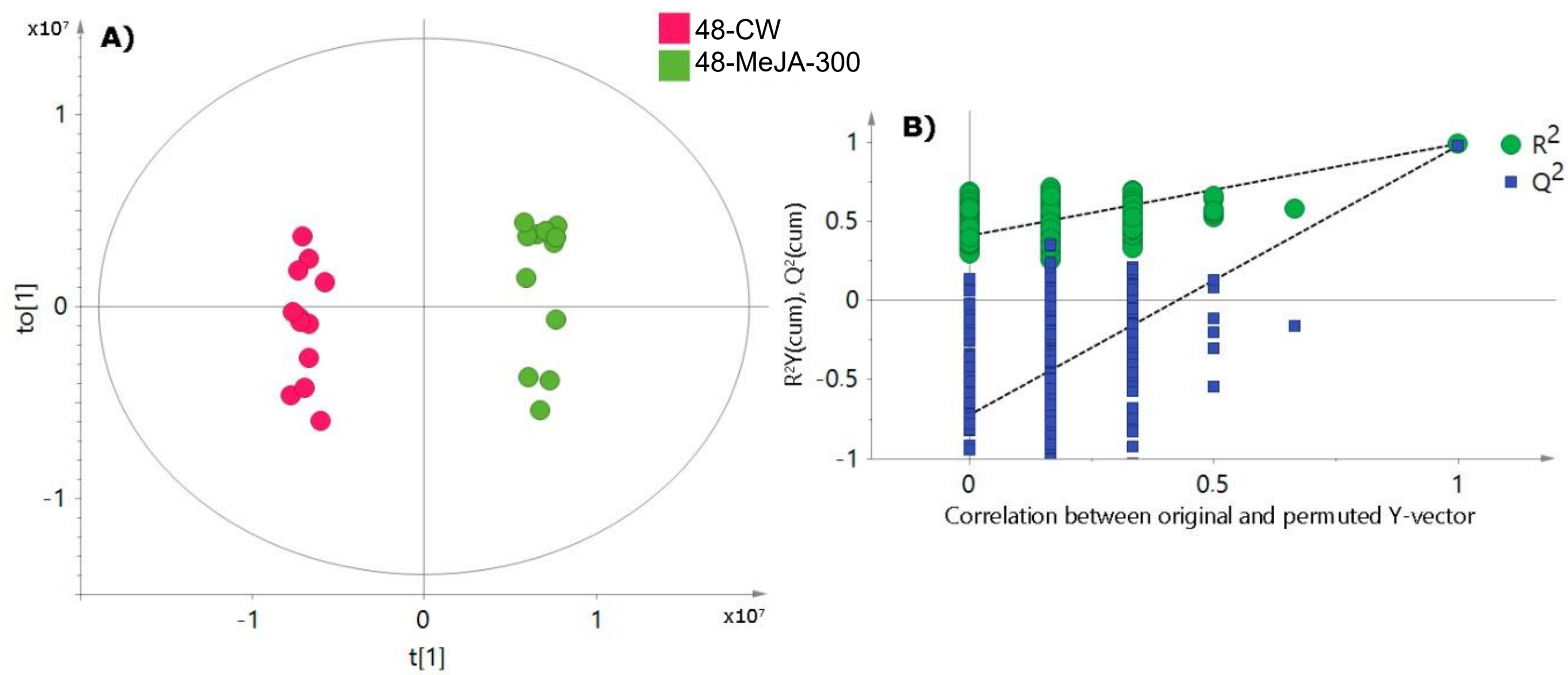

Fig S9. A) OPLS-DA-derived score plot showing the dual comparison of metabolic profiles between control (CW) and MeJA-300 treated samples at 48 hours post-treatment. **B)** Permutation test (n = 200) validating the OPLS-DA model. Permuted Q^2 and R^2Y distributions were substantially lower than original model values, and permutation regression of the Q^2 points intersects the vertical axis below zero, confirming the absence of overfitting.

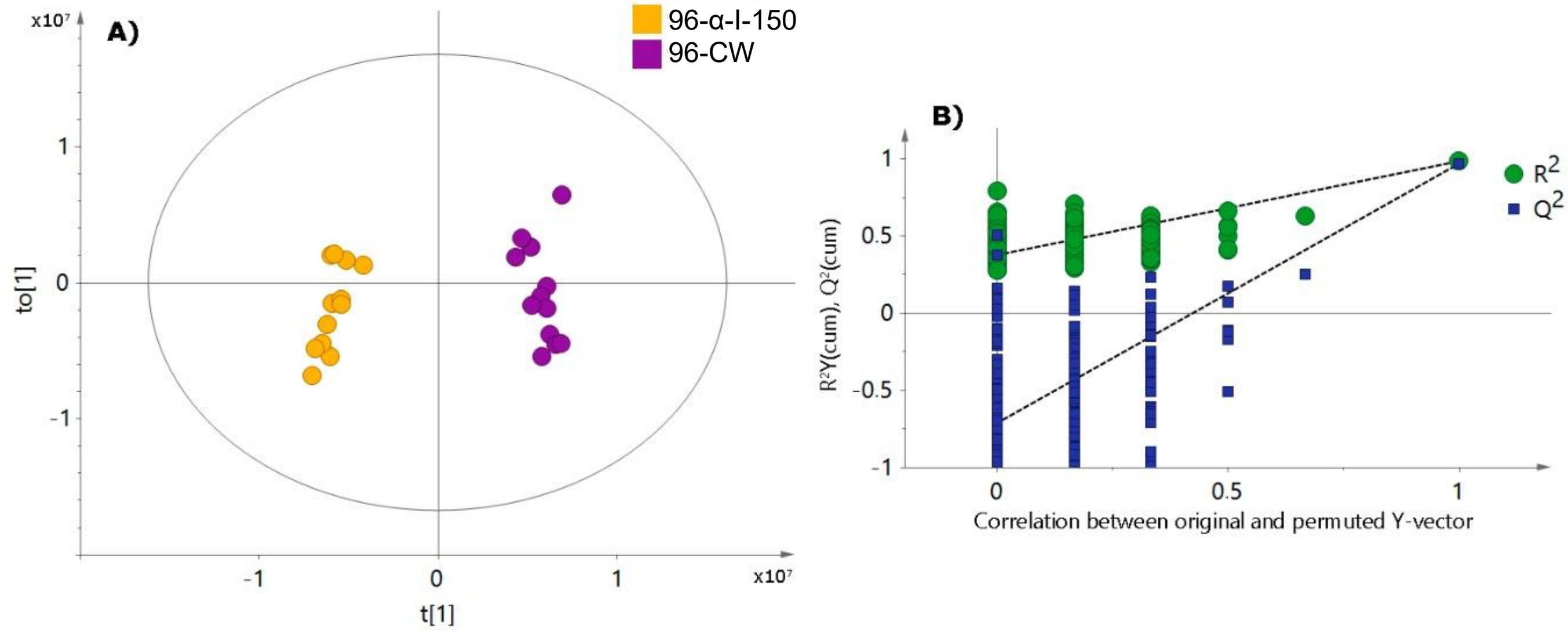

Fig S10. A) OPLS-DA-derived score plot showing the dual comparison of metabolic profiles between control (CW) and α-I-150 treated samples at 96 hours post-treatment. **B)** Permutation test (n = 200) validating the OPLS-DA model. Permuted Q^2 and R^2Y distributions were substantially lower than original model values, and permutation regression of the Q^2 points intersects the vertical axis below zero, confirming the absence of overfitting.

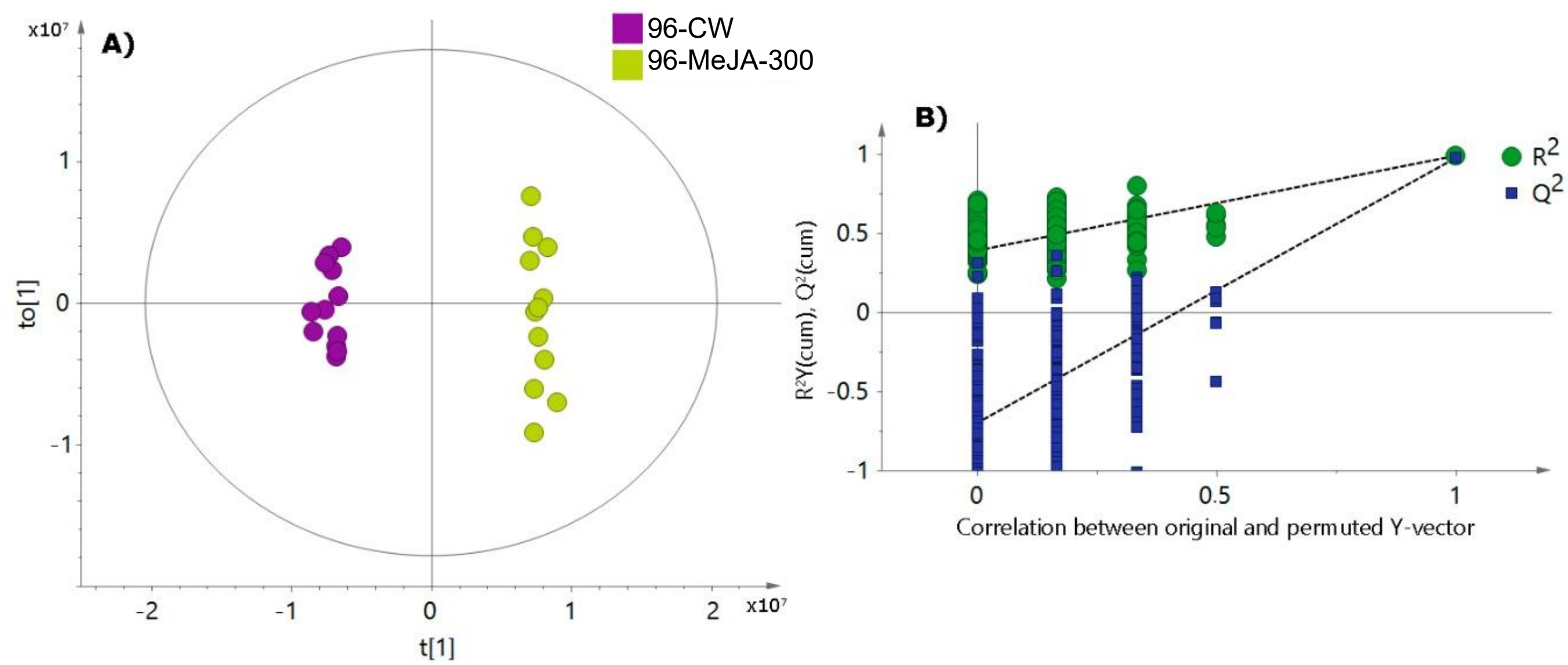

Fig S11. A) OPLS-DA-derived score plot showing the dual comparison of metabolic profiles between control (CW) and MeJA-300 treated samples at 96 hours post-treatment. **B)** Permutation test (n = 200) validating the OPLS-DA model. Permuted Q^2 and R^2Y distributions were substantially lower than original model values, and permutation regression of the Q^2 points intersects the vertical axis below zero, confirming the absence of overfitting.

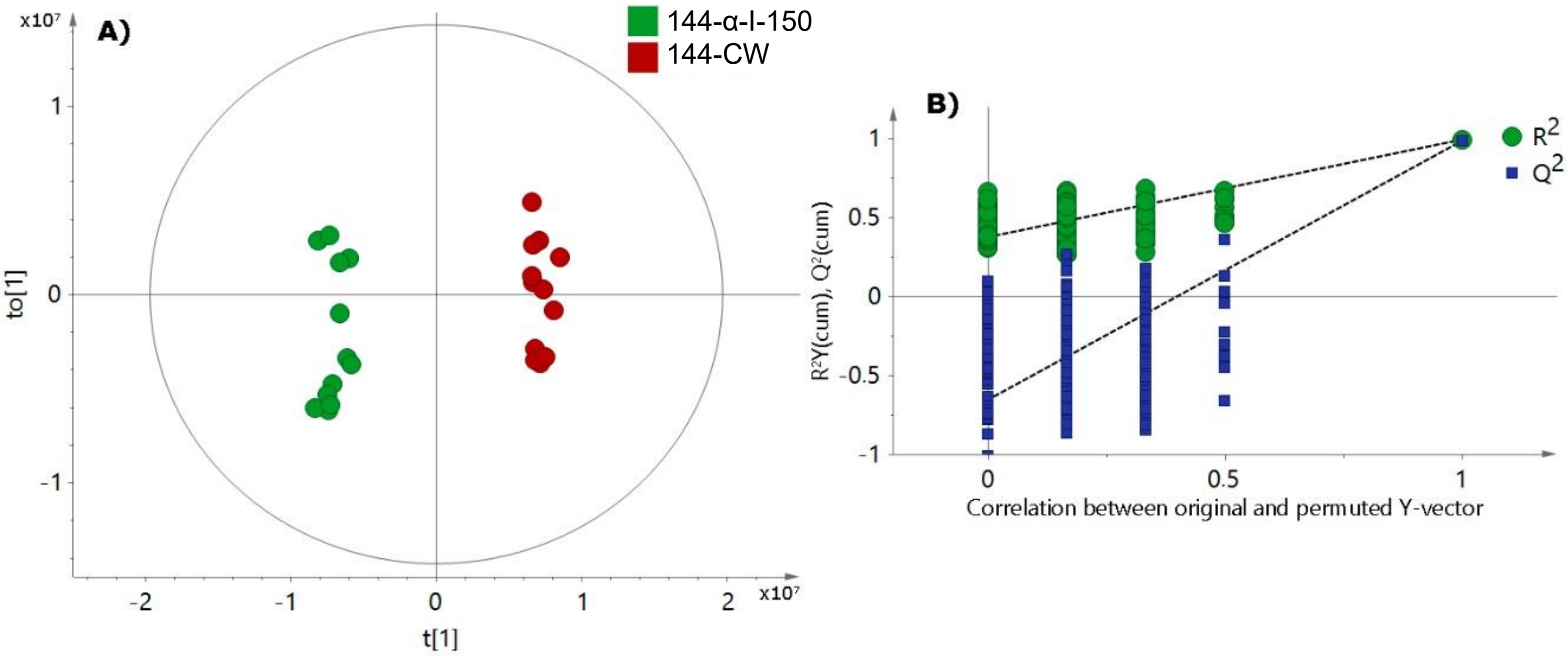

Fig S12. A) OPLS-DA-derived score plot showing the dual comparison of metabolic profiles between control (CW) and α-I-150 treated samples at 144 hours post-treatment. **B)** Permutation test (n = 200) validating the OPLS-DA model. Permuted Q^2 and R^2Y distributions were substantially lower than original model values, and permutation regression of the Q^2 points intersects the vertical axis below zero, confirming the absence of overfitting.

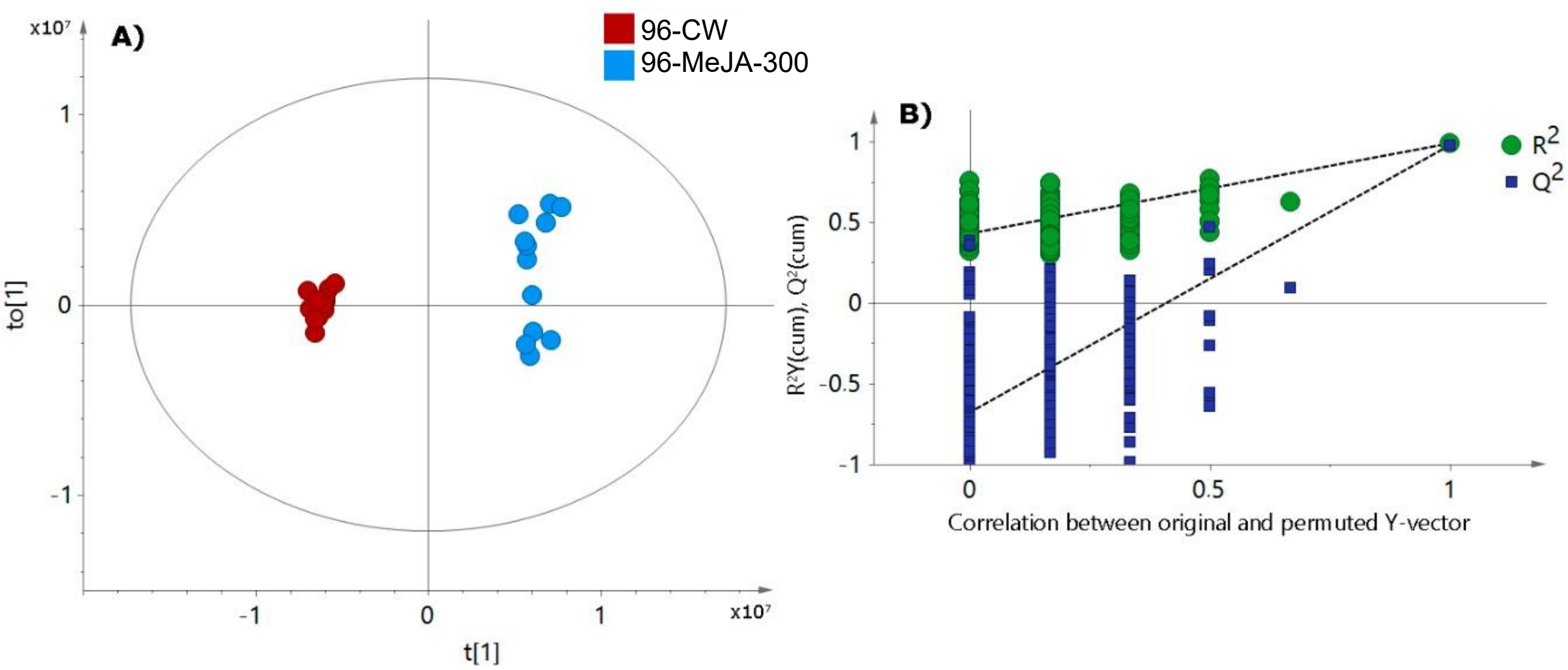

Fig S13. A) OPLS-DA-derived score plot showing the dual comparison of metabolic profiles between control (CW) and MeJA-300 treated samples at 144 hours post-treatment. **B)** Permutation test (n = 200) validating the OPLS-DA model. Permuted Q^2 and R^2Y distributions were substantially lower than original model values, and permutation regression of the Q^2 points intersects the vertical axis below zero, confirming the absence of overfitting.
