# Supplementary material for: Exogenous application of α-Ionone and Methyl jasmonate induces metabolite-based response in Alstroemeria and reduces feeding damage by Western Flower Thrips
Source: PLoS One. 2026 Aug 11;21(8):e0355314. doi: 10.1371/journal.pone.0355314 (PMC13460579; doi:10.1371/journal.pone.0355314)
Supplement: Supplementary file 2 [file pone.0355314.s002.pdf]

S2 Table. Compilation of LC-MS data of annotated metabolites in tepals and leaves of Alstroemeria treated with methyl jasmonate (MeJA) at 300 ppm and α-ionone (α-I) at 150 ppm during three post-treatment times (48-, 96-, and 144 h).

#	R_t (min) [a]	$[M+H]^+$ *m/z*	$[M–H]^-$ *m/z*	$[M–H]^-$ *m/z* HRMS teo	$[M–H]^-$ *m/z* HRMS exp	Error (ppm) [b]	Formula	Identity [c]	Metabolite class [c]
**1**	1.23	337	335	335.0767	335.0778	-3.28	$C_{16}H_{16}O_8$	caffeoylshikimic acid	Phenylpropanoid
**2**	1.24	227	225	225.0399	225.0393	2.67	$C_{10}H_{10}O_6$	chorismic acid	Phenylpropanoid
**3**	1.25	369	367	367.1029	367.1043	-3.81	$C_{17}H_{20}O_9$	feruloylquinic acid	Phenylpropanoid
**4**	1.25	225	223	223.0607	223.0617	-4.48	$C_{11}H_{12}O_5$	sinapic acid	Phenylpropanoid
**5**	1.26	469	467	467.0826	467.0841	-3.21	$C_{20}H_{20}O_{13}$	digalloylrhamnose	Benzoic acid
**6**	1.32	381	379	379.1029	379.1044	-3.96	$C_{18}H_{20}O_9$	sinapoylshikimic acid	Phenylpropanoid
**7**	1.45	311	309	309.0974	309.0982	-2.59	$C_{15}H_{18}O_7$	cinnamoylglucoside	Phenylpropanoid
**8**	1.56	315	313	313.0559	313.0572	-4.15	$C_{13}H_{14}O_9$	syringoylmalic acid 1	Benzoic acid
**9**	2.82	315	313	313.0559	313.0572	-4.15	$C_{13}H_{14}O_9$	syringoylmalic acid 2	Benzoic acid
**10**	2.90	325	323	323.1131	323.1119	3.71	$C_{16}H_{20}O_7$	dihydrocinnamoyl quinic acid	Phenylpropanoid
**11**	4.28	313	311	311.0403	311.0418	-4.82	$C_{13}H_{12}O_9$	caffeoyltartaric acid 1	Phenylpropanoid
**12**	4.69	625	623	623.1612	623.1626	-2.25	$C_{28}H_{32}O_{16}$	isorhamnetin glucoside rhamnoside 1	Flavonoid
**13**	4.70	313	311	311.0403	311.0412	-2.89	$C_{13}H_{12}O_9$	caffeoyltartaric acid 2	Phenylpropanoid
**14**	5.34	313	311	311.0556	311.0566	-3.21	$C_{17}H_{12}O_6$	acetyl-trihydroxyflavone	Flavonoid
**15**	5.38	423	421	421.0559	421.0542	4.04	$C_{22}H_{14}O_9$	hydroxybenzoylkaempferol 1	Flavonoid
**16**	5.43	625	623	623.1612	623.1626	-2.25	$C_{28}H_{32}O_{16}$	isorhamnetin glucoside rhamnoside 2	Flavonoid
**17**	5.80	571	569	569.0931	569.0943	-2.11	$C_{27}H_{22}O_{14}$	kaempferol arabinoside	Flavonoid
**18**	6.39	423	421	421.0559	421.0542	4.04	$C_{22}H_{14}O_9$	hydroxybenzoylkaempferol 2	Flavonoid
**19**	6.57	713	711	711.1409	711.1421	-1.69	$C_{30}H_{32}O_{20}$	quercetin malonyldiglucoside	Flavonoid
**20**	7.04	611	609	609.1456	609.1441	2.46	$C_{27}H_{30}O_{16}$	kaempferol diglucoside 1	Flavonoid
**21**	7.06	595	593	593.1507	593.1518	-1.85	$C_{27}H_{30}O_{15}$	quercetin dirhamnoside 1	Flavonoid
**22**	8.65	641	639	639.1197	639.1186	1.72	$C_{27}H_{28}O_{18}$	quercetin glucoside glucuronide	Flavonoid
**23**	9.23	697	695	697.198	697.1995	-2.15	$C_{31}H_{36}O_{18}$	kaempferol apiosylarabinoside rhamnoside	Flavonoid
**24**	9.40	757	755	755.2035	755.2021	1.85	$C_{33}H_{40}O_{20}$	kaempferol rutinoside glucoside	Flavonoid
**25**	9.46	611	609	609.1456	609.1439	2.79	$C_{27}H_{30}O_{16}$	kaempferol diglucoside 2	Flavonoid
**26**	9.60	465	463	463.0877	463.0862	3.24	$C_{21}H_{20}O_{12}$	quercetin glucoside 1	Flavonoid
**27**	9.74	667	665	665.2082	665.2069	1.95	$C_{31}H_{38}O_{16}$	tetramethylquercetin rutinoside	Flavonoid
**28**	9.81	451	449	449.072	449.0709	2.45	$C_{20}H_{18}O_{12}$	myricetin xyloside	Flavonoid
**29**	9.84	507	505	505.0982	505.0996	-2.77	$C_{23}H_{22}O_{13}$	quercetin acetylglucoside 1	Flavonoid
**30**	10.02	739	737	737.2293	737.2278	2.03	$C_{34}H_{40}O_{18}$	acacetin rhamnoside rutinoside	Flavonoid
**31**	10.02	693	691	691.1874	691.1858	2.31	$C_{32}H_{36}O_{17}$	methoxykaempferol bis-acetylrhamnoside	Flavonoid
**33**	10.06	449	447	447.0927	447.0944	-3.80	$C_{27}H_{44}O_5$	quercetin rhamnoside	Flavonoid
**34**	10.09	455	453	453.0458	453.0467	-1.99	$C_{22}H_{14}O_{11}$	galloylquercetin	Flavonoid
**35**	10.18	465	463	463.0877	463.0859	3.89	$C_{21}H_{20}O_{12}$	quercetin glucoside 2	Flavonoid
**36**	10.36	507	505	505.0982	505.0996	-2.77	$C_{23}H_{22}O_{13}$	quercetin acetylglucoside 2	Flavonoid
**37**	10.65	449	447	447.0927	447.0933	-1.34	$C_{21}H_{20}O_{10}$	kaempferol glucoside	Flavonoid
**38**	10.69	595	593	593.1507	593.1521	-2.36	$C_{27}H_{30}O_{15}$	quercetin dirhamnoside 2	Flavonoid
**39**	10.94	679	677	677.1718	677.1729	-1.62	$C_{31}H_{34}O_{17}$	kaempferol diacetylglucoside rhamnoside	Flavonoid
**41**	11.09	621	619	619.1663	619.1645	2.91	$C_{29}H_{32}O_{15}$	kaempferol acetyldirhamnoside	Flavonoid
**40**	11.09	533	531	531.1139	531.1121	3.39	$C_{25}H_{24}O_{13}$	kaempferol diacetylglucoside 1	Flavonoid
**42**	11.96	503	501	501.1397	501.1383	2.79	$C_{22}H_{20}O_{11}$	naringenin diacetylrhamnoside	Flavonoid
**43**	14.12	523	521	521.1295	521.1282	2.49	$C_{24}H_{26}O_{13}$	irigenin glucoside	Flavonoid
**44**	14.14	533	531	531.1139	531.1144	-0.94	$C_{25}H_{24}O_{13}$	kaempferol diacetylglucoside 2	Flavonoid
**45**	14.75	489	487	487.124	487.1254	-2.87	$C_{24}H_{24}O_{11}$	acacetin acetylglucoside	Flavonoid
**46**	14.99	557	555	555.0927	555.0941	-2.52	$C_{30}H_{20}O_{11}$	fukugetin	Flavonoid
**47**	15.08	517	515	515.1189	515.1174	2.91	$C_{25}H_{24}O_{12}$	kaempferol rhamnoside	Flavonoid
**48**	15.27	327	325	325.0712	325.0728	-4.92	$C_{18}H_{14}O_6$	dimethoxy-methylenedioxyflavone	Flavonoid

[a] Rt = retention time (min); [b] error calculated in ppm between experimental and predicted accurate (monoisotopic) mass; [c] Annotation-based identity according to accurate mass measurements and MS/MS data (annotation confidence level = 3, i.e., putative candidate for a metabolite class).
