# Supplementary material for: Exogenous application of α-Ionone and Methyl jasmonate induces metabolite-based response in Alstroemeria and reduces feeding damage by Western Flower Thrips
Source: PLoS One. 2026 Aug 11;21(8):e0355314. doi: 10.1371/journal.pone.0355314 (PMC13460579; doi:10.1371/journal.pone.0355314)
Supplement: Supplementary file 3 [file pone.0355314.s003.pdf]

S3 Table. Overview of up-/downregulated, annotated metabolites in tepals and leaves of *Alstroemeria* treated with methyl jasmonate (MeJA) at 300 ppm and α-ionone (α-I) at 150 ppm during three post-treatment times (48-, 96-, and 144 h).

			leaves [b]						tepals [b]					
**#**	**Identity [a]**	**Metabolite class [a]**	***MeJA 48***	***MeJA 96***	***MeJA 144***	***α-I 48***	***α-I 96***	***α-I 144***	***MeJA 48***	***MeJA 96***	***MeJA 144***	***α-I 48***	***α-I 96***	***α-I 144***
**1**	caffeoylshikimic acid	Phenylpropanoid							↑	↑	↑	↑	↑	
**2**	chorismic acid	Phenylpropanoid								↑	↑			
**3**	feruloylquinic acid	Phenylpropanoid	↑	↑	↑			↑						
**4**	sinapic acid	Phenylpropanoid				↓	↓	↑						
**5**	digalloylrhamnose	Benzoic acid								↓	↑		↓	↑
**6**	sinapoylshikimic acid	Phenylpropanoid	↑	↑			↑	↑						
**7**	cinnamoylglucoside	Phenylpropanoid		↑		↓	↑							
**8**	syringoylmalic acid 1	Benzoic acid							↑			↑		
**9**	syringoylmalic acid 2	Benzoic acid							↑	↓	↑	↑	↓	↑
**10**	dihydrocinnamoyl quinic acid	Phenylpropanoid							↑			↑	↑	
**11**	caffeoyltartaric acid 1	Phenylpropanoid	↑	↑	↑		↑	↑						
**12**	isorhamnetin glucoside rhamnoside 1	Flavonoid								↑	↑	↓	↑	↓
**13**	caffeoyltartaric acid 2	Phenylpropanoid										↓		
**14**	acetyl-trihydroxyflavone	Flavonoid						↑						
**15**	hydroxybenzoylkaempferol 1	Flavonoid							↑	↑			↑	↑
**16**	isorhamnetin glucoside rhamnoside 2	Flavonoid		↑	↑		↑	↑						
**17**	kaempferol arabinoside	Flavonoid							↓	↑	↑	↓	↓	↑
**18**	hydroxybenzoylkaempferol 2	Flavonoid		↑	↑	↓	↑	↑						
**19**	quercetin malonyldiglucoside	Flavonoid								↑	↑	↓		
**20**	kaempferol diglucoside 1	Flavonoid							↑	↑	↑			
**21**	quercetin dirhamnoside 1	Flavonoid							↑	↑		↓	↓	↓
**22**	quercetin glucoside glucuronide	Flavonoid								↑		↑	↑	↑
**23**	kaempferol apiosylarabinoside rhamnoside	Flavonoid									↑	↓		
**24**	kaempferol rutinoside glucoside	Flavonoid	↑	↑	↑		↑							
**25**	kaempferol diglucoside 2	Flavonoid							↑					↑
**26**	quercetin glucoside 1	Flavonoid									↑		↑	
**27**	tetramethylquercetin rutinoside	Flavonoid							↑	↑	↑	↑	↑	↑
**28**	myricetin xyloside	Flavonoid	↑	↑	↑		↑							
**29**	quercetin acetylglucoside 1	Flavonoid								↑			↑	
**30**	acacetin rhamnoside rutinoside	Flavonoid							↓	↑	↑	↑	↑	↑
**31**	methoxykaempferol bis-acetylrhamnoside	Flavonoid								↑	↑		↑	↑
**33**	quercetin rhamnoside	Flavonoid										↑	↑	
**34**	galloylquercetin	Flavonoid						↑						
**35**	quercetin glucoside 2	Flavonoid			↑			↑						
**36**	quercetin acetylglucoside 2	Flavonoid						↑						
**37**	kaempferol glucoside	Flavonoid	↑			↑								
**38**	quercetin dirhamnoside 2	Flavonoid		↑	↑	↓	↑	↑						
**39**	kaempferol diacetylglucoside rhamnoside	Flavonoid			↑	↑	↑	↑						
**41**	kaempferol acetyldirhamnoside	Flavonoid			↑			↑						
**40**	kaempferol diacetylglucoside 1	Flavonoid	↑											
**42**	naringenin diacetylrhamnoside	Flavonoid			↑	↓	↑	↑						
**43**	irigenin glucoside	Flavonoid	↑	↑	↑		↓							
**44**	kaempferol diacetylglucoside 2	Flavonoid	↑											
**45**	acacetin acetylglucoside	Flavonoid		↑		↓								
**46**	fukugetin	Flavonoid			↑	↑								
**47**	kaempferol rhamnoside	Flavonoid	↑	↑	↑	↑								
**48**	dimethoxy-methylenedioxyflavone	Flavonoid		↑		↓								

[a] Annotation-based identity according to accurate mass measurements (S2 Table) and MS/MS data (annotation confidence level = 3, i.e., putative candidate for a metabolite class); [b] Presence and regulation of VIP-based differential metabolites in *Alstroemeria* tepals and leaves under elicitor treatments and times according to the manuscript data S4 file; ↑ = upregulated; ↓ = downregulated.
