# Supplementary material for: Exogenous application of α-Ionone and Methyl jasmonate induces metabolite-based response in Alstroemeria and reduces feeding damage by Western Flower Thrips
Source: PLoS One. 2026 Aug 11;21(8):e0355314. doi: 10.1371/journal.pone.0355314 (PMC13460579; doi:10.1371/journal.pone.0355314)
Supplement: Supplementary file 4 [file pone.0355314.s004.pdf]

MANUSCRIPT DATA FOR OPLS-DA-DERIVED MODELS:

DUAL COMPARISONS (TREATMENT VS CONTROL) FOR LEAF-DERIVED SAMPLES

General list 1. Dual comparison 48-α-I-150 vs 48-CW for leaf metabolite profile data. Resulting data for OPLS-DA model ([1+1+0]; $R^2_{(cum)}$ = 0.995; $Q^2_{(cum)}$ = 0.861). VIP-based top fifteen-ranked metabolites.

Scores data			
Obs ID (Primary)	Obs ID (Class)	1,00001 * M4.t[1]	1,15633 * M4.to[1]
144-α-I-150-1	144-α-I-150	-10204000	-200850
144-α-I-150-10	144-α-I-150	-10397200	-1305330
144-α-I-150-11	144-α-I-150	-10909500	2625960
144-α-I-150-12	144-α-I-150	-10501400	1828530
144-α-I-150-2	144-α-I-150	-10179500	638708
144-α-I-150-3	144-α-I-150	-10003900	814280
144-α-I-150-4	144-α-I-150	-10948300	837119
144-α-I-150-5	144-α-I-150	-10373900	-2267260
144-α-I-150-6	144-α-I-150	-9923300	-582843
144-α-I-150-7	144-α-I-150	-10164800	-1563720
144-α-I-150-8	144-α-I-150	-10202800	-2985500
144-α-I-150-9	144-α-I-150	-10420600	2160900
48-CW-1	48-CW	10874000	2055900
48-CW-10	48-CW	9863380	2431430
48-CW-11	48-CW	11384400	-2867300
48-CW-12	48-CW	10131600	2085130
48-CW-2	48-CW	9880030	-1341230
48-CW-3	48-CW	10272800	562659
48-CW-4	48-CW	11414000	2485090
48-CW-5	48-CW	9513630	-3222990
48-CW-6	48-CW	10361700	-625641
48-CW-7	48-CW	10558600	1343150
48-CW-8	48-CW	10162700	-3807810
48-CW-9	48-CW	9812020	901603

VIP scores		
Var ID (Primary)	M4.VIP [1+1+0]	2,44693 * M4.VIP[1]cvSE
acacetin acetylglucoside	2.327	0.230
kaempferol rhamnoside	2.254	0.155
hydroxybenzoylkaempferol 2	2.192	0.169
kaempferol glucoside	1.996	0.218
cinnamoylglucoside	1.934	0.088
sinapic acid	1.778	0.162
fukugetin	1.730	0.298
dimethoxy-methylenedioxyflavone	1.644	0.144
quercetin dirhamnoside 2	1.603	0.252
kaempferol diacetylglucoside rhamnoside	1.421	0.258
naringenin diacetylrhamnoside	1.411	0.204
kaempferol diacetylglucoside 2	0.602	0.123
acetyl-trihydroxyflavone	0.495	0.206
quercetin glucoside 1	0.315	0.175
kaempferol acetyldirhamnoside	0.313	0.144

General list 2. Dual comparison 48-MeJA-300 vs 48-CW for leaf metabolite profile data. Resulting data for OPLS-DA model ([1+1+0]; $R^2_{(cum)}$ = 0.995; $Q^2_{(cum)}$ = 0.851). VIP-based top fifteen-ranked metabolites.

Scores data			
Obs ID (Primary)	Obs ID (Class)	1,00002 * M5.t[1]	1,12979 * M5.to[1]
48-CW-1	48-CW	-12645000	408909
48-CW-10	48-CW	-12658400	-165100
48-CW-11	48-CW	-12275100	390739
48-CW-12	48-CW	-13222400	-2306220
48-CW-2	48-CW	-12172900	701774
48-CW-3	48-CW	-12536700	116638
48-CW-4	48-CW	-13759800	-759175
48-CW-5	48-CW	-11744800	1141680
48-CW-6	48-CW	-12250000	698287
48-CW-7	48-CW	-12674700	197494
48-CW-8	48-CW	-12269100	1196540
48-CW-9	48-CW	-12858100	-1621560
48-MeJA-300-1	48-MeJA-300	12565800	1985350
48-MeJA-300-10	48-MeJA-300	13885200	954842
48-MeJA-300-11	48-MeJA-300	11922300	4023900
48-MeJA-300-12	48-MeJA-300	12514200	-3999250
48-MeJA-300-2	48-MeJA-300	11921300	1962240
48-MeJA-300-3	48-MeJA-300	12493300	-1982270
48-MeJA-300-4	48-MeJA-300	12891300	2315560
48-MeJA-300-5	48-MeJA-300	12529900	3341990
48-MeJA-300-6	48-MeJA-300	11602300	-1003060
48-MeJA-300-7	48-MeJA-300	13700300	519564
48-MeJA-300-8	48-MeJA-300	12552200	2138570
48-MeJA-300-9	48-MeJA-300	11853600	-2882270

VIP scores		
Var ID (Primary)	M5.VIP [1+1+0]	2,44693 * M5.VIP[1]cvSE
irigenin glucoside	2.56923	0.115219
kaempferol rhamnoside	2.47795	0.186983
caffeoyltartaric acid 1	1.92669	0.107515
sinapoylshikimic acid	1.90116	0.12927
myricetin xyloside	1.88371	0.160048
kaempferol glucoside	1.78123	0.195456
kaempferol rutinoside glucoside	1.73719	0.08803
feruloylquinic acid	1.71845	0.120111
kaempferol diacetylglucoside 2	1.63298	0.810096
kaempferol diacetylglucoside 1	1.61183	0.311161
naringenin diacetylrhamnoside	0.643747	0.275368
acacetin acetylglucoside	0.465653	0.226101
acetyl-trihydroxyflavone	0.446534	0.186786
methoxykaempferol bis-acetylrhamnoside	0.42933	0.123266
dimethoxy-methylenedioxyflavone	0.423207	0.245024

General list 3. Dual comparison 96-α-I-150 vs 96-CW for leaf metabolite profile data. Resulting data for OPLS-DA model ([1+1+0]; $R^2_{(cum)}$ = 0.998; $Q^2_{(cum)}$ = 0.885). VIP-based top fifteen-ranked metabolites.

Scores data			
Obs ID (Primary)	Obs ID (Class)	1 * M6.t[1]	1,16454 * M6.to[1]
96-α-I-150-1	96-α-I-150	-12195000	556672
96-α-I-150-10	96-α-I-150	-12728900	1717890
96-α-I-150-11	96-α-I-150	-12440900	782373
96-α-I-150-12	96-α-I-150	-12131600	-2728680
96-α-I-150-2	96-α-I-150	-12281700	1062400
96-α-I-150-3	96-α-I-150	-12005300	-1088310
96-α-I-150-4	96-α-I-150	-12463500	-1348920
96-α-I-150-5	96-α-I-150	-12494100	3014430
96-α-I-150-6	96-α-I-150	-12121800	-1869400
96-α-I-150-7	96-α-I-150	-12179700	-395296
96-α-I-150-8	96-α-I-150	-12379000	1516160
96-α-I-150-9	96-α-I-150	-13047400	-1219320
96-CW-1	96-CW	12400600	956017
96-CW-10	96-CW	12397700	-206835
96-CW-11	96-CW	12280500	2862460
96-CW-12	96-CW	12132700	-2339130
96-CW-2	96-CW	12415300	488333
96-CW-3	96-CW	12797300	-785829
96-CW-4	96-CW	12681800	957108
96-CW-5	96-CW	12709400	1766990
96-CW-6	96-CW	12329600	-729002
96-CW-7	96-CW	12309500	-576448
96-CW-8	96-CW	11809500	-346328
96-CW-9	96-CW	12205100	-2047330

VIP scores		
Var ID (Primary)	M6.VIP [1+1+0]	2,44693 * M6.VIP[1]cvSE
hydroxybenzoylkaempferol 2	1.9747	0.187714
kaempferol rutinoside glucoside	1.91241	0.131994
quercetin dirhamnoside 2	1.87091	0.167173
irigenin glucoside	1.81384	0.0810905
cinnamoylglucoside	1.76801	0.131857
caffeoyltartaric acid 1	1.69534	0.188326
sinapoylshikimic acid	1.67949	0.123725
isorhamnetin glucoside rhamnoside 2	1.62905	0.0900883
myricetin xyloside	1.6263	0.0896524
sinapic acid	1.5708	0.105241
kaempferol diacetylglucoside rhamnoside	1.4410	0.25512
naringenin diacetylrhamnoside	1.3648	0.143197
feruloylquinic acid	0.53297	0.307741
quercetin rhamnoside	0.30990	0.387547
kaempferol acetyldirhamnoside	0.28716	0.348316

General list 4. Dual comparison 96-MeJA-300 vs 96-CW for leaf metabolite profile data. Resulting data for OPLS-DA model ([1+1+0]; $R^2_{(cum)}$ = 0.996; $Q^2_{(cum)}$ = 0.954). VIP-based top fifteen-ranked metabolites.

Scores data			
Obs ID (Primary)	Obs ID (Class)	1 * M7.t[1]	1,12966 * M7.to[1]
96-CW-1	96-CW	-14656500	688274
96-CW-10	96-CW	-14949300	-1309840
96-CW-11	96-CW	-14396200	2906650
96-CW-12	96-CW	-14881900	-2189020
96-CW-2	96-CW	-14646300	793981
96-CW-3	96-CW	-14660900	-379543
96-CW-4	96-CW	-15166300	-1267560
96-CW-5	96-CW	-14475500	1561050
96-CW-6	96-CW	-14530800	-455079
96-CW-7	96-CW	-14857900	-1381770
96-CW-8	96-CW	-14689900	841809
96-CW-9	96-CW	-14528900	191047
96-MeJA-300-1	96-MeJA-300	15127800	-661085
96-MeJA-300-10	96-MeJA-300	15276200	-3812280
96-MeJA-300-11	96-MeJA-300	15587700	-371976
96-MeJA-300-12	96-MeJA-300	14239400	-2041200
96-MeJA-300-2	96-MeJA-300	14576500	992694
96-MeJA-300-3	96-MeJA-300	14683900	115744
96-MeJA-300-4	96-MeJA-300	14325400	950892
96-MeJA-300-5	96-MeJA-300	15584200	2834460
96-MeJA-300-6	96-MeJA-300	14223100	-189028
96-MeJA-300-7	96-MeJA-300	14183800	3985120
96-MeJA-300-8	96-MeJA-300	14452100	3836830
96-MeJA-300-9	96-MeJA-300	14180200	-2940180

VIP scores		
Var ID (Primary)	M7.VIP [1+1+0]	2,44693 * M7.VIP[1]cvSE
quercetin dirhamnoside 2	2.0413	0.106364
caffeoyltartaric acid 1	1.89802	0.10121
myricetin xyloside	1.82606	0.163094
hydroxybenzoylkaempferol 2	1.78033	0.162238
kaempferol rutinoside glucoside	1.77516	0.0937156
cinnamoylglucoside	1.69166	0.0994169
sinapoylshikimic acid	1.61046	0.103583
dimethoxy-methylenedioxyflavone	1.60076	0.123749
feruloylquinic acid	1.54888	0.103942
isorhamnetin glucoside rhamnoside 2	1.52402	0.0736497
acacetin acetylglucoside	1.49479	0.0878694
irigenin glucoside	1.31556	0.269249
kaempferol rhamnoside	1.28634	0.287412
kaempferol diacetylglucoside 2	0.595667	0.257481
fukugetin	0.548446	0.22837

General list 5. Dual comparison 144-α-I-150 vs 144-CW for leaf metabolite profile data. Resulting data for OPLS-DA model ([1+1+0]; $R^2_{(cum)}$ = 0.998; $Q^2_{(cum)}$ = 0.954). VIP-based top twenty-ranked metabolites.

Scores data			
Obs ID (Primary)	Obs ID (Class)	M8.t[1]	1,08861 * M8.to[1]
144-α-I-150-1	144-α-I-150	-15691900	270074
144-α-I-150-10	144-α-I-150	-15091400	1546840
144-α-I-150-11	144-α-I-150	-15657100	1427010
144-α-I-150-12	144-α-I-150	-15388400	183853
144-α-I-150-2	144-α-I-150	-15525800	-471053
144-α-I-150-3	144-α-I-150	-15425900	-1873450
144-α-I-150-4	144-α-I-150	-16020200	-1085460
144-α-I-150-5	144-α-I-150	-15550700	2846500
144-α-I-150-6	144-α-I-150	-15737900	-2390110
144-α-I-150-7	144-α-I-150	-15770800	3387250
144-α-I-150-8	144-α-I-150	-15286000	-1279540
144-α-I-150-9	144-α-I-150	-15187700	-2561920
144-CW-1	144-CW	15424300	-122553
144-CW-10	144-CW	15310300	-612217
144-CW-11	144-CW	15748400	451756
144-CW-12	144-CW	15459300	52542.8
144-CW-2	144-CW	15336600	-591583
144-CW-3	144-CW	15304600	-737171
144-CW-4	144-CW	15503800	493588
144-CW-5	144-CW	15571600	288996
144-CW-6	144-CW	15774700	565998
144-CW-7	144-CW	15716000	110287
144-CW-8	144-CW	15681900	411590
144-CW-9	144-CW	15502400	-311232

VIP scores		
Var ID (Primary)	M8.VIP [1+1+0]	2,44693 * M8.VIP[1]cvSE
kaempferol acetyldirhamnoside	1.71401	0.0880507
naringenin diacetylrhamnoside	1.43223	0.0782138
kaempferol diacetylglucoside rhamnoside	1.3952	0.108108
isorhamnetin glucoside rhamnoside 2	1.37758	0.0571198
quercetin dirhamnoside 2	1.34787	0.107536
galloylquercetin	1.29919	0.0582953
sinapoylshikimic acid	1.29387	0.090901
acetyl-trihydroxyflavone	1.27957	0.0759518
hydroxybenzoylkaempferol 2	1.24069	0.0973231
quercetin acetylglucoside 2	1.2221	0.0709785
quercetin glucoside 2	1.22158	0.109294
sinapic acid	1.21902	0.0970717
feruloylquinic acid	1.16958	0.0776884
caffeoyltartaric acid 1	1.16547	0.0968508
irigenin glucoside	0.34893	0.0862372
digalloylrhamnose	0.266232	0.148123
quercetin rhamnoside	0.219963	0.178931
quercetin glucoside 1	0.18869	0.188254
methoxykaempferol bis-acetylrhamnoside	0.180756	0.166299
quercetin acetylglucoside 1	0.152513	0.130472

General list 6. Dual comparison 144-MeJA-300 vs 144-CW for leaf metabolite profile data. Resulting data for OPLS-DA model ([1+1+0]; $R^2_{(cum)}$ = 0.996; $Q^2_{(cum)}$ = 0.945). VIP-based top twenty-ranked metabolites.

Scores data			
Obs ID (Primary)	Obs ID (Class)	1 * M9.t[1]	1,14055 * M9.to[1]
144-CW-1	144-CW	-18908300	526352
144-CW-10	144-CW	-19342600	-602846
144-CW-11	144-CW	-18980200	777856
144-CW-12	144-CW	-19134800	-126984
144-CW-2	144-CW	-18960800	555576
144-CW-3	144-CW	-19012500	501597
144-CW-4	144-CW	-18981400	288865
144-CW-5	144-CW	-19549300	-1003510
144-CW-6	144-CW	-19190600	586460
144-CW-7	144-CW	-19302600	-19372.8
144-CW-8	144-CW	-19578700	-986888
144-CW-9	144-CW	-19423000	-497103
144-MeJA-300-1	144-MeJA-300	19988300	-2896210
144-MeJA-300-10	144-MeJA-300	18858400	-3184030
144-MeJA-300-11	144-MeJA-300	20088000	804277
144-MeJA-300-12	144-MeJA-300	20150900	1857110
144-MeJA-300-2	144-MeJA-300	18177600	1232840
144-MeJA-300-3	144-MeJA-300	19464800	2314940
144-MeJA-300-4	144-MeJA-300	18417900	-2302540
144-MeJA-300-5	144-MeJA-300	18377500	3007980
144-MeJA-300-6	144-MeJA-300	19340700	2061220
144-MeJA-300-7	144-MeJA-300	18491600	-1177950
144-MeJA-300-8	144-MeJA-300	20374100	-489664
144-MeJA-300-9	144-MeJA-300	18634900	3972020

VIP scores		
Var ID (Primary)	M9.VIP [1+1+0]	2,44693 * M9.VIP[1]cvSE
kaempferol acetyldirhamnoside	1.55178	0.109504
quercetin dirhamnoside 2	1.4763	0.0820227
kaempferol rhamnoside	1.4622	0.0517079
kaempferol diacetylglucoside rhamnoside	1.37113	0.113032
irigenin glucoside	1.34532	0.143369
kaempferol rutinoside glucoside	1.23796	0.0661334
naringenin diacetylrhamnoside	1.22536	0.144772
feruloylquinic acid	1.20497	0.136057
quercetin glucoside 2	1.2033	0.0691018
caffeoyltartaric acid 1	1.20078	0.068119
isorhamnetin glucoside rhamnoside 2	1.18459	0.118841
fukugetin	1.17699	0.142223
hydroxybenzoylkaempferol 2	1.16045	0.0622742
myricetin xyloside	1.15734	0.0975509
quercetin glucoside 1	0.43989	0.0988241
quercetin rhamnoside	0.42742	0.107922
quercetin acetylglucoside 2	0.40038	0.0459365
galloylquercetin	0.36378	0.063364
methoxykaempferol bis-acetylrhamnoside	0.32588	0.122417
kaempferol diacetylglucoside 2	0.267866	0.209301

MANUSCRIPT DATA FOR OPLS-DA-DERIVED MODELS:

DUAL COMPARISONS (TREATMENT VS CONTROL) FOR TEPAL-DERIVED SAMPLES

General list 7. Dual comparison 48-α-I-150 vs 48-CW for tepal metabolite profile data. Resulting data for OPLS-DA model ([1+1+0]; $R^2_{(cum)}$ = 0.975; $Q^2_{(cum)}$ = 0.699). VIP-based top twenty-ranked metabolites.

Scores data			
Obs ID (Primary)	Obs ID (Class)	1,00023 * M4.t[1]	1,17442 * M4.to[1]
48-α-I-150-1	48-α-I-150	-5628410	-1019960
48-α-I-150-10	48-α-I-150	-6232420	-2107090
48-α-I-150-11	48-α-I-150	-6892670	1608320
48-α-I-150-12	48-α-I-150	-5729980	-4804710
48-α-I-150-2	48-α-I-150	-5785850	2118380
48-α-I-150-3	48-α-I-150	-5465400	-3531490
48-α-I-150-4	48-α-I-150	-5852870	-1575570
48-α-I-150-5	48-α-I-150	-5475120	2044590
48-α-I-150-6	48-α-I-150	-6813280	-3652240
48-α-I-150-7	48-α-I-150	-6259460	-128031
48-α-I-150-8	48-α-I-150	-5082340	3103970
48-α-I-150-9	48-α-I-150	-5926730	-3756170
48-CW-1	48-CW	6409000	3206760
48-CW-10	48-CW	5526060	2230300
48-CW-11	48-CW	5058670	-4108300
48-CW-12	48-CW	6462090	3414340
48-CW-2	48-CW	6554210	-2222250
48-CW-3	48-CW	5142580	2702760
48-CW-4	48-CW	5203150	1936080
48-CW-5	48-CW	5588490	-2575810
48-CW-6	48-CW	6789580	2894860
48-CW-7	48-CW	5856290	1882160
48-CW-8	48-CW	7436350	-664220
48-CW-9	48-CW	5518060	2403310

VIP scores		
Var ID (Primary)	M4.VIP [1+1+0]	2,44693 * M4.VIP[1]cvSE
kaempferol arabinoside	3.4991	0.949824
kaempferol apiosylarabinoside rhamnoside	1.82601	0.893042
isorhamnetin glucoside rhamnoside 1	1.69392	0.857574
quercetin dirhamnoside 1	1.57256	0.648442
caffeoylshikimic acid	1.48066	1.91304
tetramethylquercetin rutinoside	1.47264	0.569114
quercetin glucoside glucuronide	1.31964	0.696029
syringoylmalic acid 1	1.29386	0.360745
quercetin malonyldiglucoside	1.2673	0.222499
acacetin rhamnoside rutinoside	1.2127	1.34975
dihydrocinnamoyl quinic acid	1.20874	0.945545
caffeoyltartaric acid 2	1.14781	0.791138
syringoylmalic acid 2	1.12654	0.521291
quercetin rhamnoside	1.10119	0.400183
sinapic acid	0.784046	0.295436
quercetin glucoside 1	0.783604	0.57555
kaempferol diacetylglucoside rhamnoside	0.770057	0.663585
kaempferol diglucoside 1	0.663866	0.549288
methoxykaempferol bis-acetylrhamnoside	0.557466	0.404033
quercetin glucoside 2	0.490461	0.600198

General list 8. Dual comparison 48-MeJA-300 vs 48-CW for tepal metabolite profile data. Resulting data for OPLS-DA model ([1+1+0]; $R^2_{(cum)}$ = 0.978; $Q^2_{(cum)}$ = 0.753). VIP-based top twenty-ranked metabolites.

Scores data			
Obs ID (Primary)	Obs ID (Class)	1,00026 * M5.t[1]	1,30422 * M5.to[1]
48-CW-1	48-CW	-6725650	-2691260
48-CW-10	48-CW	-7744940	-4639310
48-CW-11	48-CW	-6197410	1349720
48-CW-12	48-CW	-7260530	-590995
48-CW-2	48-CW	-6718330	2574520
48-CW-3	48-CW	-6703750	-937114
48-CW-4	48-CW	-6956690	-4278920
48-CW-5	48-CW	-7086180	3773140
48-CW-6	48-CW	-7175620	-811389
48-CW-7	48-CW	-6377110	-5444040
48-CW-8	48-CW	-7324070	1993290
48-CW-9	48-CW	-7633820	-297654
48-MeJA-300-1	48-MeJA-300	6578800	3773630
48-MeJA-300-10	48-MeJA-300	7592730	3326160
48-MeJA-300-11	48-MeJA-300	7786100	4226870
48-MeJA-300-12	48-MeJA-300	7704930	-688570
48-MeJA-300-2	48-MeJA-300	7094900	3936340
48-MeJA-300-3	48-MeJA-300	6057890	-4354830
48-MeJA-300-4	48-MeJA-300	7725400	3569830
48-MeJA-300-5	48-MeJA-300	5978180	1498460
48-MeJA-300-6	48-MeJA-300	6789470	-5813580
48-MeJA-300-7	48-MeJA-300	6029170	3664960
48-MeJA-300-8	48-MeJA-300	5821960	4389600
48-MeJA-300-9	48-MeJA-300	7344570	-4528850

VIP scores		
Var ID (Primary)	M5.VIP [1+1+0]	2,44693 * M5.VIP[1]cvSE
hydroxybenzoylkaempferol 1	4.06397	0.347663
caffeoylshikimic acid	2.15199	1.73629
kaempferol arabinoside	1.45096	1.4399
acacetin rhamnoside rutinoside	1.41449	1.0486
quercetin dirhamnoside 1	1.28852	0.910397
kaempferol diglucoside 1	1.28098	0.329027
dihydrocinnamoyl quinic acid	1.2618	0.859993
syringoylmalic acid 1	1.23598	0.373913
kaempferol diglucoside 2	1.22094	0.918712
tetramethylquercetin rutinoside	1.15832	1.02241
syringoylmalic acid 2	1.11509	1.2282
caffeoyltartaric acid 2	0.734019	1.08746
acetyl-trihydroxyflavone	0.715178	0.654866
sinapic acid	0.682067	0.326037
quercetin glucoside 1	0.63719	0.619554
quercetin dirhamnoside 1	0.522735	0.679392
kaempferol diacetylglucoside rhamnoside	0.504728	0.208252
kaempferol glucoside	0.490147	0.188395
methoxykaempferol bis-acetylrhamnoside	0.421635	0.254375
caffeoyltartaric acid 1	0.421485	0.158064

General list 9. Dual comparison 96-α-I-150 vs 96-CW for tepal metabolite profile data. Resulting data for OPLS-DA model ([1+1+0]; $R^2_{(cum)}$ = 0.967; $Q^2_{(cum)}$ = 0.797). VIP-based top twenty-ranked metabolites.

Scores data			
Obs ID (Primary)	Obs ID (Class)	1,001 * M6.t[1]	1,12574 * M6.to[1]
96-α-I-150-1	96-α-I-150	-5922210	-1521740
96-α-I-150-10	96-α-I-150	-6206720	-3100270
96-α-I-150-11	96-α-I-150	-4531920	1504990
96-α-I-150-12	96-α-I-150	-6004820	-5419970
96-α-I-150-2	96-α-I-150	-5139030	1890140
96-α-I-150-3	96-α-I-150	-6436040	-4465400
96-α-I-150-4	96-α-I-150	-5368850	-1282780
96-α-I-150-5	96-α-I-150	-5950040	2248560
96-α-I-150-6	96-α-I-150	-6849040	-4863290
96-α-I-150-7	96-α-I-150	-5416820	-1647490
96-α-I-150-8	96-α-I-150	-5805390	2325760
96-α-I-150-9	96-α-I-150	-7048880	-6868510
96-CW-1	96-CW	6304310	-3799350
96-CW-10	96-CW	6606760	-4547430
96-CW-11	96-CW	4353950	2295660
96-CW-12	96-CW	6147720	-274957
96-CW-2	96-CW	5237720	3030660
96-CW-3	96-CW	5792250	-1059300
96-CW-4	96-CW	5830470	-5402750
96-CW-5	96-CW	4715660	3736790
96-CW-6	96-CW	6145160	-1912430
96-CW-7	96-CW	6898620	-4478870
96-CW-8	96-CW	6961730	5787620
96-CW-9	96-CW	5285410	-1675640

VIP scores		
Var ID (Primary)	M6.VIP [1+1+0]	2,44693 * M6.VIP[1]cvSE
acacetin rhamnoside rutinoside	2.42178	0.399206
hydroxybenzoylkaempferol 1	2.26314	0.342788
quercetin glucoside 1	2.2319	0.498415
isorhamnetin glucoside rhamnoside 1	1.98803	0.458634
caffeoylshikimic acid	1.84482	0.55868
quercetin glucoside glucuronide	1.81383	1.17037
quercetin rhamnoside	1.54375	0.382601
tetramethylquercetin rutinoside	1.54358	1.28172
methoxykaempferol bis-acetylrhamnoside	1.46432	0.913551
quercetin acetylglucoside 1	1.383104	0.557439
kaempferol arabinoside	1.348482	0.458356
syringoylmalic acid 2	1.323324	0.245992
dihydrocinnamoyl quinic acid	1.243299	0.429917
digalloylrhamnose	1.234206	0.53944
quercetin dirhamnoside 1	1.134348	0.172741
acetyl-trihydroxyflavone	0.629012	0.167111
kaempferol glucoside	0.615383	0.257008
quercetin malonyldiglucoside	0.525653	0.219501
sinapic acid	0.508971	0.384773
feruloylquinic acid	0.506868	0.246767

General list 10. Dual comparison 96-MeJA-300 vs 96-CW for tepal metabolite profile data. Resulting data for OPLS-DA model ([1+1+0]; $R^2_{(cum)}$ = 0.975; $Q^2_{(cum)}$ = 0.798). VIP-based top twenty-ranked metabolites.

Scores data			
Obs ID (Primary)	Obs ID (Class)	1,00041 * M7.t[1]	1,14179 * M7.to[1]
96-CW-1	96-CW	-7077940	2438090
96-CW-10	96-CW	-6383470	4051640
96-CW-11	96-CW	-7374230	-3290010
96-CW-12	96-CW	-8419420	-2029520
96-CW-2	96-CW	-7286780	-2512420
96-CW-3	96-CW	-7564370	-482774
96-CW-4	96-CW	-7287720	3468670
96-CW-5	96-CW	-7366820	-3972270
96-CW-6	96-CW	-8575940	-652968
96-CW-7	96-CW	-7606980	2951030
96-CW-8	96-CW	-7327920	-3599470
96-CW-9	96-CW	-6632170	430014
96-MeJA-300-1	96-MeJA-300	7420140	-631212
96-MeJA-300-10	96-MeJA-300	8021210	293947
96-MeJA-300-11	96-MeJA-300	7982550	4776050
96-MeJA-300-12	96-MeJA-300	8070450	-5124540
96-MeJA-300-2	96-MeJA-300	7137110	7593780
96-MeJA-300-3	96-MeJA-300	7344920	-7898940
96-MeJA-300-4	96-MeJA-300	7645550	-347533
96-MeJA-300-5	96-MeJA-300	7248240	5187100
96-MeJA-300-6	96-MeJA-300	7372470	-9696610
96-MeJA-300-7	96-MeJA-300	7615890	-3486420
96-MeJA-300-8	96-MeJA-300	7037350	3539480
96-MeJA-300-9	96-MeJA-300	8507880	-8905130

VIP scores		
Var ID (Primary)	M7.VIP [1+1+0]	2,44693 * M7.VIP[1]cvSE
kaempferol arabinoside	2.62022	0.678804
quercetin acetylglucoside 1	2.43123	0.41023
hydroxybenzoylkaempferol 1	2.36037	0.445604
isorhamnetin glucoside rhamnoside 1	1.87792	0.47558
tetramethylquercetin rutinoside	1.66514	0.723526
quercetin glucoside glucuronide	1.52967	0.907162
quercetin malonyldiglucoside	1.51363	0.920302
acacetin rhamnoside rutinoside	1.45738	0.700841
methoxykaempferol bis-acetylrhamnoside	1.42847	0.783882
caffeoylshikimic acid	1.42695	0.748821
syringoylmalic acid 2	1.37967	0.278198
chorismic acid	1.37859	0.204184
kaempferol diglucoside 1	1.32819	0.299713
quercetin dirhamnoside 1	1.27793	0.219434
digalloylrhamnose	1.16093	0.492722
sinapic acid	0.54413	0.341557
quercetin malonyldiglucoside	0.46912	0.115279
kaempferol glucoside	0.45642	0.333185
cinnamoylglucoside	0.451497	0.20797
hydroxybenzoylkaempferol 2	0.417904	0.222718

General list 11. Dual comparison 144-α-l-150 vs 144-CW for tepal metabolite profile data. Resulting data for OPLS-DA model ([1+1+0]; $R^2_{(cum)}$ = 0.983; $Q^2_{(cum)}$ = 0.741). VIP-based top twenty-ranked metabolites.

Scores data			
Obs ID (Primary)	Obs ID (Class)	1,00022 * M8.t[1]	1,15724 * M8.to[1]
144-α-l-150-1	144-α-l-150	-7124670	-4765100
144-α-l-150-10	144-α-l-150	-7440000	-6151060
144-α-l-150-11	144-α-l-150	-8363310	-6048270
144-α-l-150-12	144-α-l-150	-8245090	3456000
144-α-l-150-2	144-α-l-150	-7495470	-5347210
144-α-l-150-3	144-α-l-150	-7490920	3715400
144-α-l-150-4	144-α-l-150	-6098730	-3374780
144-α-l-150-5	144-α-l-150	-7269080	-5871240
144-α-l-150-6	144-α-l-150	-6159690	2503470
144-α-l-150-7	144-α-l-150	-7527290	-990760
144-α-l-150-8	144-α-l-150	-5849410	-3705220
144-α-l-150-9	144-α-l-150	-6810230	2278720
144-CW-1	144-CW	6685270	638415
144-CW-10	144-CW	7391950	271797
144-CW-11	144-CW	7182230	-3681640
144-CW-12	144-CW	8543100	1940980
144-CW-2	144-CW	6783330	-2873680
144-CW-3	144-CW	7113690	2849660
144-CW-4	144-CW	8049690	-825662
144-CW-5	144-CW	6784750	-3485170
144-CW-6	144-CW	6631680	2645450
144-CW-7	144-CW	6591570	978560
144-CW-8	144-CW	7498800	-3345580
144-CW-9	144-CW	6617850	4886870

VIP scores		
Var ID (Primary)	M8.VIP [1+1+0]	2,44693 * M8.VIP[1]cvSE
methoxykaempferol bis-acetylrhamnoside	2.99346	0.546345
tetramethylquercetin rutinoside	2.15781	1.24126
digalloylrhamnose	2.1385	0.797543
kaempferol diglucoside 2	1.80016	0.84709
acacetin rhamnoside rutinoside	1.64729	0.937174
quercetin glucoside glucuronide	1.62338	0.813213
quercetin dirhamnoside 1	1.50573	0.613834
isorhamnetin glucoside rhamnoside 1	1.44442	0.613167
kaempferol arabinoside	1.39241	0.350394
syringoylmalic acid 2	1.33963	0.172636
hydroxybenzoylkaempferol 1	1.295732	0.340466
quercetin malonyldiglucoside	0.879361	0.123146
quercetin acetylglucoside 1	0.848415	0.452404
feruloylquinic acid	0.821386	0.430098
kaempferol apiosylarabinoside rhamnoside	0.729319	0.275466
quercetin rhamnoside	0.691032	0.331185
myricetin xyloside	0.638437	0.345153
kaempferol acetylglucoside	0.600762	0.255709
sinapic acid	0.537909	0.128416
kaempferol glucoside	0.535142	0.324484

General list 12. Dual comparison 144-MeJA-300 vs 144-CW for tepal metabolite profile data. Resulting data for OPLS-DA model ([1+1+0]; $R^2_{(cum)}$ = 0.980; $Q^2_{(cum)}$ = 0.791). VIP-based top twenty-ranked metabolites.

Scores data			
Obs ID (Primary)	Obs ID (Class)	1,00014 * M9.t[1]	1,19917 * M9.to[1]
144-CW-1	144-CW	-6018240	-281207
144-CW-10	144-CW	-5978070	147569
144-CW-11	144-CW	-5799220	823068
144-CW-12	144-CW	-6997340	696349
144-CW-2	144-CW	-6407520	-661148
144-CW-3	144-CW	-5959510	332215
144-CW-4	144-CW	-6893170	-205633
144-CW-5	144-CW	-6102010	55206.3
144-CW-6	144-CW	-6464600	249142
144-CW-7	144-CW	-6577210	-1479580
144-CW-8	144-CW	-6586920	-792898
144-CW-9	144-CW	-5377680	1116920
144-MeJA-300-1	144-MeJA-300	6084650	-3205740
144-MeJA-300-10	144-MeJA-300	7289620	-2371800
144-MeJA-300-11	144-MeJA-300	6781130	4307980
144-MeJA-300-12	144-MeJA-300	5884720	2588730
144-MeJA-300-2	144-MeJA-300	5715890	888070
144-MeJA-300-3	144-MeJA-300	5211180	4735960
144-MeJA-300-4	144-MeJA-300	6253840	-1935950
144-MeJA-300-5	144-MeJA-300	5704860	3111840
144-MeJA-300-6	144-MeJA-300	7064410	5294420
144-MeJA-300-7	144-MeJA-300	5858270	-2574310
144-MeJA-300-8	144-MeJA-300	7741790	5143290
144-MeJA-300-9	144-MeJA-300	5571130	3317500

VIP scores		
Var ID (Primary)	M9.VIP [1+1+0]	2,44693 * M9.VIP[1]cvSE
methoxykaempferol bis-acetylrhamnoside	2.77367	0.820322
tetramethylquercetin rutinoside	2.55394	1.25279
digalloylrhamnose	2.43468	0.56424
caffeoylshikimic acid	2.08685	0.658
acacetin rhamnoside rutinoside	2.05031	0.793588
syringoylmalic acid 2	1.98329	0.154584
kaempferol apiosylarabinoside rhamnoside	1.76441	0.522747
quercetin glucoside 1	1.69096	0.514609
kaempferol diglucoside 1	1.65194	0.321628
isorhamnetin glucoside rhamnoside 1	1.51825	0.496131
kaempferol arabinoside	1.49852	0.669072
chorismic acid	1.47131	0.353467
quercetin malonyldiglucoside	1.37047	0.15177
kaempferol acetylglucoside	0.750114	0.445005
quercetin rhamnoside	0.730508	0.370592
quercetin dirhamnoside 2	0.708848	0.955527
sinapic acid	0.692166	0.352681
quercetin glucoside glucuronide	0.607284	0.460875
dihydrocinnamoyl quinic acid	0.597544	0.967445
caffeoyltartaric acid 2	0.522917	0.308963
